# Patterns and Drivers of Pest and Disease Occurrence in UK Treescapes

**DOI:** 10.1111/gcb.70706

**Published:** 2026-02-06

**Authors:** Peter S. Stewart, Louise J. Barwell, Katharine Turvey, Jane Barbrook, Sarah Green, Ana Pérez‐Sierra, Bethan V. Purse, Daniel Chapman

**Affiliations:** ^1^ School of Mathematics and Statistics University of Glasgow Glasgow UK; ^2^ Biological and Environmental Sciences University of Stirling Stirling UK; ^3^ UK Centre for Ecology and Hydrology Wallingford UK; ^4^ Animal and Plant Health Agency Plant Health and Seeds Inspectorate York UK; ^5^ Forest Research Northern Research Station Roslin UK; ^6^ Forest Research Alice Holt Lodge Farnham UK

**Keywords:** causal inference, Forest, integrated species distribution model, invasive species, newLEAF, pathogen, pest and disease, pest monitoring

## Abstract

Tree pests and diseases are a key threat to woodland biodiversity and commercial forestry worldwide. In the UK, the ongoing spread of pests and diseases is severely affecting a range of nationally important tree species, resulting in substantial ecological and economic impacts. As the risk posed by pests and diseases varies across the UK's treescapes, understanding the patterns of risk and the factors underlying these patterns is crucial for designing and implementing effective mitigation strategies. To address this challenge, we modelled the distribution of pests and diseases across mainland Great Britain, focusing on the total pest and disease burdens for nine host tree species of particular ecological, economic and cultural importance. Using integrated species distribution models, we combined two datasets—totalling 18,871 pest and disease records across 22 years—to model the spatial patterns of risk. To examine the factors underlying these distributions, we used graph‐based causal inference approaches to inform our model design and to explore the robustness of our conclusions to variations in our modelling assumptions. We found that pest and disease burdens for broadleaved host trees exhibited hotspots in England, while burdens for conifer hosts tended to be high in Scotland. We identified urban area, human population density and local recreation as important drivers for several species, mainly native broadleaves. By contrast, woodland connectivity, afforestation and the level of conifer coverage were the most important drivers of pest and disease burdens for conifer hosts. Deforestation was also an important driver, with effects on pest and disease burdens for both conifers and broadleaves. Our findings have implications for the management of the UK's treescapes in the face of continuing threats from tree pests and diseases, including supporting targeted surveillance and the prioritisation of tree species for future planting.

## Graphical abstract

Tree pests and diseases can be very damaging to natural and commercial forests. We studied how the risk of tree pests and diseases varies across mainland Great Britain and explored how factors such as urbanisation and recreational visits affect the level of risk in different places.

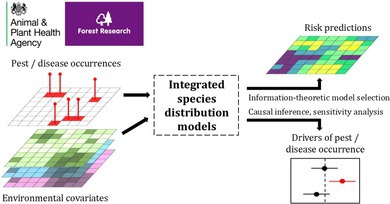

## 1 Introduction

Tree diseases and insect pests are an important constituent of forest ecosystems (Mordecai 2011). However, outbreaks of these species can be highly destructive, with major impacts on forest biodiversity and ecosystem services (Boyd et al. 2013; Freer‐Smith and Webber 2017). Furthermore, anthropogenic global change is significantly altering the distributions and dynamics of tree pests and diseases (Ramsfield et al. 2016; Simler‐Williamson et al. 2019). In many cases, global change is driving outbreaks that are more frequent and severe. For example, land‐use and climate change can cause outbreaks of native pests and diseases (Burgess et al. 2022), while increasing global connectivity through trade leads to pests and diseases being transported across biogeographic boundaries, driving outbreaks of introduced species (Roy et al. 2014). Numerous examples illustrate the ecological and economic damage caused by recent outbreaks. For instance, in the UK, ash dieback (caused by the fungus *Hymenoscyphus fraxineus*) is expected to incur economic costs of £14.8 billion over the next century (Hill et al. 2019) and is predicted to place 45 obligate ash‐associated species at risk of extirpation (Mitchell et al. 2022). As global change continues to intensify, tree pests and diseases are likely to become an increasingly prevalent threat to forests (Potter and Urquhart 2017). For instance, projections of future tree pest and disease invasion rates in the UK suggest that severe impacts on a range of nationally important tree species could occur (Bebber et al. 2025). Preparedness for these threats is increasingly prioritised within national and regional biosecurity and tree and forest health policies (e.g., Scottish Plant Health Strategy, Tree Health Resilience Strategy, Plant Biosecurity Strategy for Great Britain, EC Priority Pest regulations).

The risk posed to forests by pests and diseases is not spatially homogeneous, but rather varies at several spatial scales. At an international scale, patterns of risk are heavily influenced by trade (Roy et al. 2014), alongside other factors including the richness of available hosts and phylogenetic distance between host species (Gilbert and Webb 2007; Gougherty and Davies 2022) and the traits of the pest or pathogen (Barwell et al. 2021). These findings, as well as the difficulty of containing an outbreak following introduction (Potter and Urquhart 2017; Roy et al. 2014), underscore the importance of biosecurity (Roy et al. 2014) supported by horizon scanning to anticipate emerging threats (Barwell et al. 2021). However, it is also important to understand spatial heterogeneity in pest and disease risk at a finer scale. Early detection of pest or disease outbreaks significantly improves the feasibility of eradication or containment and the cost‐effectiveness of biosecurity interventions (Jones and Kleczkowski 2020). Predictions of pest and disease risk within a country can improve awareness of pest and disease threats and allow management efforts and often limited surveillance resources to be allocated to high‐risk areas. For example, spatial risk frameworks for *Phytophthora ramorum* risk to Larch and heathland fragments informed surveillance by Forestry Commission Scotland (now Scottish Forestry) between 2012 and 2017 (Purse et al. 2016).

Estimating the effect of factors which drive pest and disease risk is also important; a range of environmental and anthropogenic factors can potentially influence the probability that a pest or pathogen is introduced and subsequently establishes in an area, such as land cover and connectivity (Ellis et al. 2010), human population density and associated urbanisation (Colunga‐Garcia et al. 2010), and the prevalence of specific activities such as commercial forestry (Jules et al. 2002) or recreation (Hall et al. 2020). Understanding the relative importance of different drivers enables biosecurity and management efforts to be targeted at specific drivers with the greatest potential to reduce pest and disease impacts (e.g., public engagement campaigns to improve biosecurity among recreationists; Hall et al. 2020). Understanding the pest and disease burdens on different forest types is also relevant for better integration of biosecurity risks into decision‐making on tree species selection and assisted migration to improve the resilience of future forests to threats from pests and diseases (Conway and Vander Vecht 2015; Ennos et al. 2019) as well as climate change.

Species distribution models provide a powerful tool to help understand the patterns and drivers of pest and disease risk. However, several challenges inhibit the creation of useful models. One key challenge is the fundamental tension between prediction and inference; models which are selected to produce good predictions—using methods like information criteria (e.g., AIC; Akaike 1973) and cross‐validation (Stone 1977) —run a substantial risk of producing inaccurate inferences of the effects of explanatory variables because they often favour models which exhibit included‐variable bias (i.e., when a covariate confounds the focal effect estimate; Arif and MacNeil 2022; Cinelli et al. 2020; McElreath 2021; Stewart et al. 2023). Consequently, predicting the patterns of pest and disease occurrence and inferring their drivers call for different approaches. One approach to the inference problem is to use directed acyclic graphs (DAGs) to represent mechanistic assumptions about the interactions between explanatory variables, and how these variables directly and indirectly affect species occurrence (Greenland et al. 1999; Pearl 1995). The DAG can then be analysed to obtain a set of covariates which will allow for an accurate estimate of the effect of a given focal driver variable (Greenland et al. 1999; Pearl 1995). The structure of the DAG can then be modified to examine the sensitivity of this estimated effect to varying the mechanistic assumptions.

A second challenge in modelling pest and disease occurrence is the quality of the available data. The responsibility for pest and disease monitoring may be divided across multiple organisations, with individual organisations only covering a subset of the focal geographic area or pest and disease species (Barwell et al. 2025). Furthermore, the quantity of data held in any individual dataset may not be sufficient to obtain precise predictions and effect estimates. To overcome these issues, it would be ideal to synthesise all available data within a single model. However, doing so introduces additional difficulties; the available data may be collected using different methods and may be of entirely different types (e.g., presence‐absence vs. presence‐background data). Integrated species distribution models (ISDMs) provide a solution to this problem.

Commonly, ISDMs use a single underlying spatial point process model to represent the true species distribution and then assign separate observation models to each set of observed data (Miller et al. 2019; Mostert and O'Hara 2023). Recent developments have facilitated the fitting of these hierarchical state‐space ISDMs within a Bayesian framework using integrated nested Laplace approximation (Bachl et al. 2019; Lindgren and Rue 2015; Mostert and O'Hara 2023; Rue et al. 2009). This approach bears several advantages. First, these models can incorporate spatial random effects in a computationally efficient manner using the stochastic partial differential equation (SPDE) approximation (Lindgren et al. 2011). This computational efficiency means that multiple fields can be included within a single model, which can improve the model's ability to deal with spatially biased presence‐background data (Simmonds et al. 2020). Second, the Bayesian approach enables the use of regularising prior distributions, which can mitigate overfitting and improve predictive performance (McElreath 2021). In particular, the use of penalised complexity priors (Simpson et al. 2017) for the spatial random effects ensures that the model intrinsically favours a lack of spatial structure, meaning that such structure will be absent unless informed by the observed data. However, despite the strength of Bayesian ISDMs, to our knowledge they have not yet been applied to modelling the distributions of tree pests and diseases.

Here, we use Bayesian ISDMs to model the distributions of tree pests and diseases in mainland Great Britain. We focus total pest and disease burdens for a set of widespread and abundant host tree species with particular ecological, economic and cultural significance in the UK. Our study has two main objectives: (1) predict the relative intensity of pest and disease occurrence for each host tree species; (2) estimate the effects of eight potential drivers—recreation, area of urban land, human population, afforestation, deforestation, distance to the nearest border control post, area of conifer forest and woodland connectivity—on the intensity of pest and disease occurrence. We approach this second aim through the use of graph‐based causal inference approaches, allowing us to examine the sensitivity of our effect estimates to varying our modelling assumptions. Our findings are informative for the design and implementation of plant health interventions, such as targeted surveillance and the prioritisation of tree species for future planting.

## 2 Methods

### 2.1 Data

We used two spatially referenced datasets on pest and disease occurrence in mainland Great Britain. The first dataset, obtained from the Animal and Plant Health Agency (APHA), contained records of inspections made by plant health inspectors to a range of premises throughout England and Wales (similar data were not available at a sufficient spatial resolution for Scotland), sampling plant material from 19 priority tree genera widely planted or trialled in the UK landscape (see Supporting Methods). As our focus was on pest and disease occurrence in the wider environment, we subset the data to retain only inspections of woodland, heathland, watercourses, farms, gardens (registered parks and gardens, e.g., the grounds of stately homes, not private gardens attached to residential properties) or recreational facilities (including many public parks). We further subset the data to cases where the plant group was identified as grown plants or growing crops, or the job description was provided as ‘established plantings’, in order to exclude seed inspections. After subsetting, 17,147 inspection records remained, with the date of inspection ranging from 31/05/2000 to 29/12/2022. We grouped observations by location and visit date; if at least one pest was detected on a given visit date, this counted as a presence for the location. As many locations were visited on multiple dates, the data comprise a count of detections out of a total count of surveys for each location. In total, 1995 locations were visited, with the number of visits per location ranging from 1 to 81 (Q1 = 1, median = 1, Q3 = 2). The second dataset contained records from Forest Research's Tree Health Diagnostic and Advisory Service (THDAS), covering England, Wales and Scotland. These records comprise reports made by members of the public (including by letter, email, telephone and the Forest Research TreeAlert online reporting tool), with species identity subsequently confirmed by experts at Forest Research, primarily using molecular methods (for microbial pathogens) and morphology (for insect pests). As these data do not contain observations where no pest was found, they are presence‐background data. We subset the THDAS data to observations made after the year 2000 to ensure consistency with the APHA dataset. Following subsetting, 1724 THDAS observations remained.

We also obtained a range of environmental variables which may be associated with pest and disease occurrence. We derived the area and edge length per 1 km grid cell of woodland (all types), broadleaf forest and conifer forest using National Forest Inventory data (Forest Research 2023) and urban/suburban land from the UK Centre for Ecology and Hydrology (UKCEH) 10 m land cover map for the year 2020 (Morton et al. 2021). The woodland/forest area and edge variables were included to capture information on woodland size and configuration, while urban area was included because pest and disease prevalence often follows an urban–rural gradient (Branco et al. 2019; Colunga‐Garcia et al. 2010). As connectivity can influence the dynamics of pest and disease spread (Ellis et al. 2010; Huang et al. 2024; Purse et al. 2016), we derived connectivity layers for the three woodland/forest layers by using the *focal* function in the *terra* package (Hijmans 2024) to calculate a weighted sum of the values of each layer in the cells surrounding each grid cell, using a Gaussian distance function with standard deviation = 3 (i.e., 68% of the weight is assigned to cells ≤ 3 km and 99.7% assigned to cells ≤ 9 km from the focal cell). As ancient woodlands may have different levels of vulnerability to pests and diseases, for instance due to their composition or level of visitation by humans, Ancient Woodland Inventory data for England, Wales and Scotland (Natural England 2023; Natural Resources Wales 2021; NatureScot 2022) were used to derive the total area of ancient woodland in each grid cell. Afforestation and deforestation can influence pest and disease spread, for instance via propagule introduction on machinery (Jules et al. 2002) or planted plants (Donald et al. 2021; Dunn et al. 2021). To obtain information on the distribution of afforestation and deforestation, we used the UKCEH land cover change 25 m 1990–2015 dataset (Rowland et al. 2020) to calculate the proportion of each 1 km grid cell that had changed to and from woodland (any type) respectively. Maps of predicted vascular plant α‐diversity (species richness), derived from models fitted to data from 170,272 georeferenced vegetation plots, were obtained from Sabatini et al. (2022). These data were included because areas with higher plant diversity may have more primary or secondary hosts available for pests and pathogens. Canopy height, which may influence microclimatic conditions or act as a proxy for forest composition or maturity, was obtained from Lang et al. (2023). Human population density could potentially affect pest and disease spread, for example due to the movement of propagules on footwear or vehicles. We used human population data from the Natural Environment Research Council Environmental Information Data Centre (NERC EIDC), which is based on the 2011 census (Reis et al. 2017). To capture additional information about the accessibility of different areas to humans and vehicles, we calculated the total length of roads in each grid cell using the OS Open Roads dataset (Ordnance Survey 2022). Dispersal by recreationists (e.g., on footwear or sporting equipment) is a potential pathway for pest and disease spread in the UK (Hall et al. 2019). To incorporate information on the distribution of recreation activities, we used weekly and yearly recreation demand maps from Ridding et al. (2023), which contain the predicted number of visits to an area for non‐vehicular recreation (e.g., walking, cycling). The weekly recreation data were included to capture information on the footfall arising from relatively local and routine visits to an area, while the yearly data capture relatively long‐distance and/or duration visits (e.g., during holidays). In addition to attracting recreationists, parks and gardens may influence pest and disease spread due to the high prevalence of non‐native plants which could act as hosts and the risk of propagules being introduced during ornamental planting. Consequently, parks and gardens may act as sources for pests and diseases to spill over into surrounding areas (Barham et al. 2016; Potter and Urquhart 2017; Wondafrash et al. 2021). We obtained data on the location of registered parks and gardens for England, Scotland and Wales (Historic England 2023; Historic Environment Scotland 2023; The Welsh Historic Environment Service (Cadw) 2023) and calculated the distance from each grid cell to the nearest park/garden. Border control posts (BCPs), where plants and plant products entering the UK are inspected, could potentially act as pest and disease sources if propagules on imported produce escape into the wider environment. We obtained BCP locations from the Department for Environment, Food and Rural Affairs (Defra) Plant Health Information Portal (Defra 2024) and calculated the distance from each grid cell to the nearest BCP. As some tree pathogens are waterborne and can be spread in rivers (e.g., Corcobado et al. 2023), river and flow accumulation (accumulated area) layers were obtained from the HydroRIVERS and HydroSHEDS databases respectively (Lehner et al. 2008; Lehner and Grill 2013). Vapour pressure deficit (VPD; the difference between level of water vapour in the air and the level of water vapour at which the air is saturated; Novick et al. 2024) can influence outbreak dynamics by affecting microclimate suitability for pests and pathogens and by causing physiological stress for host trees (Mosedale et al. 2024; Novick et al. 2024; Romero et al. 2022). We obtained monthly vapour pressure deficit (VPD) data from 2000 to 2022 from the Centre for Environmental Data Analysis (CEDA) archive (Met Office et al. 2022), summing the monthly values to obtain a total VPD value for each grid cell. Elevation acts as a proxy for other climatic variables (e.g., temperature) and may capture information about other factors such as accessibility; we used elevation data derived from the Copernicus Land Monitoring Service (2016). Finally, we obtained a polygon of mainland Great Britain, with the islands removed, from Dambly et al. (2023). We omitted islands from our analysis as their pest and disease distributions are likely to be governed by different processes—such as the probability of dispersal from the mainland—than distributions on the mainland. We also omitted Northern Ireland from our analysis, as its pest and disease distributions are likely to be closely linked to those of the Republic of Ireland, for which we did not have data.

We used the British National Grid coordinate reference system (EPSG: 27700), with the units converted to kilometres, throughout. We processed all covariate data to obtain raster layers across England, Wales and Scotland with 1 km resolution and standardised all layers by subtracting the mean and dividing by the standard deviation. All analyses were conducted in R version 4.4.0 (R Core Team 2024). Code to reproduce our analyses is available at: https://doi.org/10.5281/zenodo.15641235

### 2.2 Patterns of Pest and Disease Occurrence

To predict the spatial patterns of pests and diseases, we fitted integrated species distribution models (ISDMs) using the *PointedSDMs* package (v.1.3.2, Mostert and O'Hara 2023). This package builds upon the *R‐INLA* (v.24.05.01‐1, Lindgren and Rue 2015; Rue et al. 2009) and *inlabru* (v.2.10.1, Bachl et al. 2019) packages to fit models in a Bayesian framework using integrated nested Laplace approximation (INLA). We fitted separate models for nine priority host species, which had sufficient wider environment data available for modelling: sycamore ( *Acer pseudoplatanus* ), silver birch ( *Betula pendula* ), common beech ( *Fagus sylvatica* ), ash ( *Fraxinus excelsior* ), English oak ( *Quercus robur* ), rowan ( *Sorbus aucuparia* ), Norway spruce ( *Picea abies* ), Sitka spruce ( *Picea sitchensis* ) and Scots pine ( *Pinus sylvestris* ).

The ISDMs combine the presence‐absence and presence‐background data by assuming a single underlying process model, which is a log‐Gaussian Cox process (Mostert and O'Hara 2023). The response variable in the process model is the intensity (i.e., the expected abundance of points at a given location; Mostert and O'Hara 2023). Each dataset then has a separate observation model: the presence‐absence data have a binomial model with a complementary log–log (cloglog; Kéry and Royle 2016) link function, while the presence‐background data have a thinned Poisson model (Mostert and O'Hara 2023).

The shared process model incorporates spatial autocorrelation by including a Gaussian random field with a Matérn covariance function (Bachl et al. 2019; Mostert and O'Hara 2023). Additionally, we included a second Gaussian random field in the observation model for the presence‐background data, as doing so can improve predictive accuracy when information on the spatial bias in the data is not available (Simmonds et al. 2020). These random fields are approximated using the stochastic partial differential equation (SPDE) approach, in which the continuous field is approximated using a discrete triangular mesh (Lindgren et al. 2011). As predictions can be sensitive to the resolution of this mesh (Dambly et al. 2023), we fitted five sets of models with different maximum edge values: 5, 10, 20, 30 and 40 km. In all cases, we assigned a minimum allowed distance of 1 km between mesh nodes to prevent the mesh from being constructed at a finer scale than our spatial covariates. We constructed all meshes using the *fmesher* package (Lindgren 2023). We used penalised complexity priors (Simpson et al. 2017) with *p*(*σ* > 1) = 0.05 and *p*(*ρ* < 10 km) = 0.05, where *σ* and *ρ* are the standard deviation and range of the Matérn covariance function respectively. We used normal priors with a mean of 0 and precision of 1 for all fixed effects and intercepts.

We selected covariates for the ISDM using an information‐theoretic approach, in which we compared models using the widely applicable information criterion (WAIC; Watanabe 2010). However, WAIC is not suitable for use in point process models, and a reliable implementation of the deviance information criterion (Spiegelhalter et al. 2002) does not currently exist for point process models in INLA. Consequently, we instead fitted binomial generalised linear models to the presence‐absence data and then compared these models using WAIC. These models incorporated a Gaussian random field and used the same prior distributions as in the ISDMs. We generated a candidate set of models for each host species by randomly sampling from our set of covariates: we sampled from 5 to 20 covariates per model, with 50 models per number of covariates, for a total of 800 candidate models per species. We then used the covariates from the top‐performing model for each host species in the respective ISDM. We argue that this approach is reasonable because the presence‐absence and presence‐background data are assumed to share the same underlying process model (Mostert and O'Hara 2023). We report the covariates used for each ISDM in Table S1. We produced maps of the predicted pest and disease intensity at 5 km resolution. Following previous literature (Morera‐Pujol et al. 2023), we rescaled the intensity to a 0–1 range, with higher values representing a greater level of pest and disease occurrence. We then rescaled the standard deviation of the predictions to match the rescaled mean.

To investigate the distribution of predicted pest and disease intensity relative to the distribution of each host tree species, we used forest sub‐compartments data (Forestry Commission 2016; Natural Resources Wales 2025) which contain percentage cover values for the primary, secondary and tertiary component tree species in 237,077 forest sub‐compartments. We used these data to obtain the percentage cover of each grid cell in our predicted intensity maps covered by each host tree species. We then modelled intensity as a function of host species cover, using robust regression (implemented with the *MASS* package; Venables and Ripley 2002) to reduce the influence of outliers and plotted the residuals to identify areas where intensity is higher or lower than expected for the level of host species cover.

### 2.3 Drivers of Pest and Disease Occurrence

We explored the effects of eight potential key drivers—recreation, urban area, human population, afforestation, deforestation, distance to the nearest border control post, conifer area and woodland connectivity—on spatial patterns in pest and disease intensity. For recreation, we further examined the effects of weekly and yearly recreation separately. We considered these variables because they have the potential to directly or indirectly affect the probability that a pest or disease is introduced to an area, subsequently establishes, or both (see Supporting Methods). We modelled the effects of each driver separately for the nine host tree species listed above.

**FIGURE 1:**
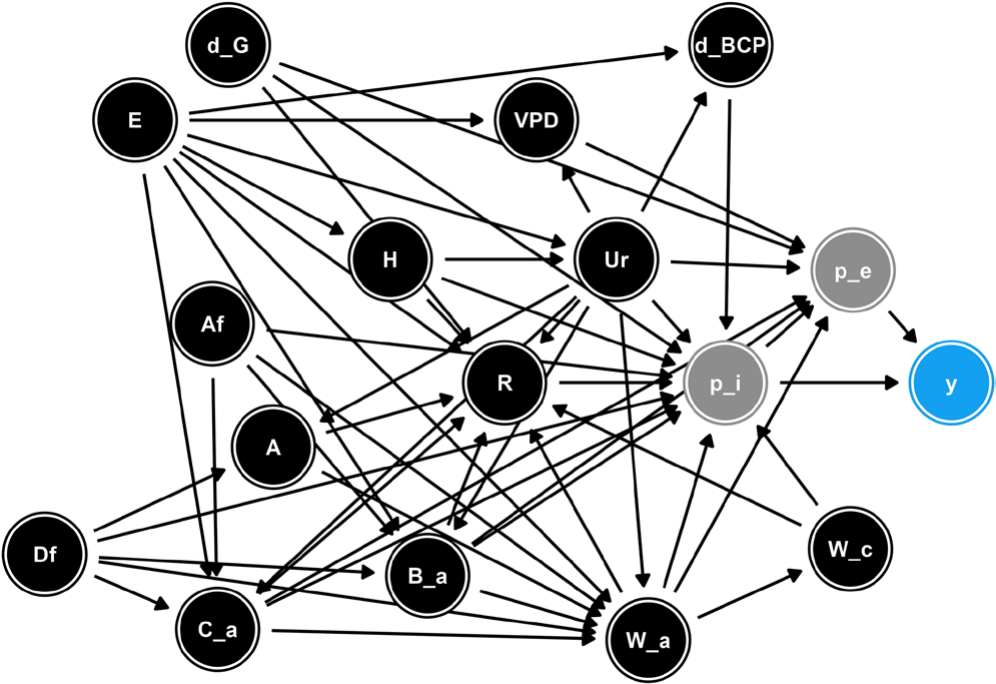
Directed acyclic graph (DAG) representing our assumptions about the data‐generating process. Nodes represent variables, while arrows represent assumed functional links. We assumed that pest and disease occurrence (y) is a function of the probability that it is introduced to a location (p_i) and that it subsequently establishes (p_e), which are latent variables. These probabilities are affected, directly or indirectly, by a range of other variables: Elevation (E), distance to park/garden (d_G), distance to border control post (d_BCP), vapour pressure deficit (VPD), human population (H), urban area (Ur), afforestation (Af), deforestation (Df), ancient woodland area (A), conifer area (C_a), broadleaf area (B_a), woodland area (W_a), woodland connectivity (W_c) and recreation (R).

We used a graph‐based causal inference approach (Greenland et al. 1999; Pearl 1995) to select variables for our models. We first encoded our assumptions about the data‐generating process in a directed acyclic graph (DAG; Figure 1); we provide a full explanation of these assumptions in the Supporting Methods. In this stage, we focused on a subset of 14 explanatory variables which included our eight focal drivers, along with a further six variables (elevation, VPD, distance to park/garden, woodland area, broadleaf area and ancient woodland area) which were deemed likely, based on the literature (see Supporting Methods), to mediate and/or confound the focal effects. We then used the *dagitty* package (Textor et al. 2016) to analyse this DAG and obtain the minimum adjustment set (i.e., the set of variables which satisfy the back‐door criterion; Pearl 1995) required to identify the total effect of each focal variable. We fitted a separate model for each focal variable, including the respective minimum adjustment set as covariates. All models were ISDMs, with the same spatial random effects and prior distributions as for the predictive models described above. As the process model in the ISDM is a log‐Gaussian Cox process (Mostert and O'Hara 2023), we assumed that the log‐intensity is linearly related to the covariates and that the effect of each covariate is monotonic.

To explore whether our inferences were robust to our assumptions about the data‐generating process, we conducted a sensitivity analysis on the DAG structure. For each focal variable, we constructed a sequence of DAGs beginning with the focal variable alone (i.e., the unadjusted effect estimate) and adding additional variables one at a time until the maximal DAG (Figure 1) was reached. We then obtained the minimum adjustment set for each DAG and ran all unique models which these sets implied. In cases where a single DAG implied multiple minimum adjustment sets, we ran all sets as separate models. Furthermore, to check whether our inferences were sensitive to the choice of mesh used for the spatial effects, we fitted models using maximum edge lengths of 5 and 30 km.

## 3 Results

Across the nine host tree species in our sample, the total number of pest and disease records ranged widely, with varying degrees of balance between the presence‐background and presence‐absence data (Table 1). Four species— *A. pseudoplatanus* , *B. pendula* , *F. sylvatica* and *F. excelsior* —had many more presence‐absence than presence‐background observations. By contrast, two species— *P. abies* and *P. sitchensis* —had many more presence‐background observations. The other three species— *P. sylvestris* , *Q. robur* and *S. aucuparia* —had relatively balanced numbers of presence‐background and presence‐absence observations. Within the presence‐absence data, the median number of surveys per site was one for all species (Table 1). However, all species had some sites that were visited multiple times, with the mean number of visits ranging from 1.04 for *S. aucuparia* to 2.22 for *P. abies* . Most surveys did not result in a detection, with all species having a median of zero detections. However, all species other than *P. sitchensis* had at least one presence‐absence detection, with the mean number of detections per site being highest for *P. abies* (mean = 0.13).

**TABLE 1:** Number of presence‐background (PB) and presence‐absence (PA) observations for each host tree species, with summary statistics (mean, maximum) for the number of surveys and detections per visited site for the presence‐absence data.

Species	Total observations		Surveys		Detections	
	PB	PA	Mean	Max.	Mean	Max.
*Acer pseudoplatanus*	55	214	1.46	53	0.05	4
*Betula pendula*	37	167	1.11	7	0.03	1
*Fagus sylvatica*	130	1113	1.57	25	0.02	2
*Fraxinus excelsior*	183	642	1.17	20	0.10	2
*Quercus robur*	502	587	1.27	6	0.03	2
*Sorbus aucuparia*	58	46	1.04	2	0.11	1
*Picea abies*	128	32	2.22	19	0.13	1
*Picea sitchensis*	137	23	1.17	3	0.00	0
*Pinus sylvestris*	157	111	1.22	5	0.06	1

### 3.1 Patterns of Pest and Disease Occurrence

**FIGURE 2:**
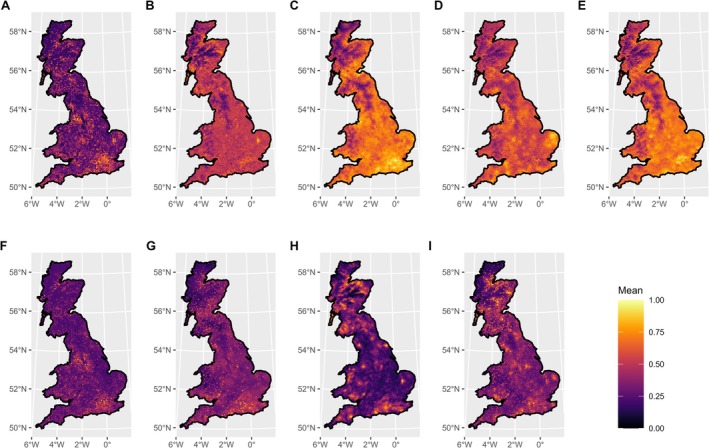
Predicted mean intensity of pest and disease occurrence, rescaled to a range of 0–1, for (A) *Acer pseudoplatanus* , (B) *Betula pendula* , (C) *Fagus sylvatica* , (D) *Fraxinus excelsior* , (E) *Quercus robur* , (F) *Sorbus aucuparia* , (G) *Picea abies* , (H) *Picea sitchensis* and (I) *Pinus sylvestris* . Predictions were generated from an ISDM using a maximum mesh edge length of 5 km. For model covariates, see Table S1. Map lines delineate study areas and do not necessarily depict accepted national boundaries.

**FIGURE 3:**
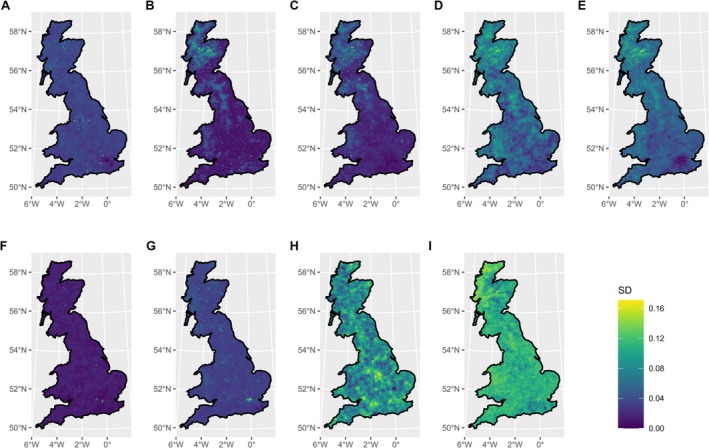
Standard deviation of predicted pest and disease occurrence intensity, rescaled to match the 0–1 scale used for the mean, for (A) *Acer pseudoplatanus* , (B) *Betula pendula* , (C) *Fagus sylvatica* , (D) *Fraxinus excelsior* , (E) *Quercus robur* , (F) *Sorbus aucuparia* , (G) *Picea abies* , (H) *Picea sitchensis* and (I) *Pinus sylvestris* . Predictions were generated from an ISDM using a maximum mesh edge length of 5 km. For model covariates, see Table S1. Map lines delineate study areas and do not necessarily depict accepted national boundaries.

Our models predicted that the intensity of pest and disease occurrence is spatially variable throughout mainland Britain (Figure 2). Several of the broadleaved species— *A. pseudoplatanus* , *F. sylvatica* , *Q. robur* and *S. aucuparia* —exhibited pest and disease hotspots in parts of England, particularly around London, Liverpool and Manchester (Figures 2A,C,E,F). *Fraxinus excelsior* also displayed hotspots in several parts of England, with a particularly large hotspot in East Anglia (Figure 2D). By contrast, predicted pest and disease occurrence for *B. pendula* was greatest in south‐western and north‐eastern Scotland, although some hotspots in south‐eastern England were still present (Figure 2B). Two of the conifer species— *P. sitchensis* and *P. sylvestris* —displayed qualitatively similar patterns, with large areas of high predicted intensity across Scotland and numerous smaller hotspots in England and Wales (Figures 2H,I). The other conifer, *P. abies* , had less pronounced hotspots, with the highest areas of predicted pest and disease intensity being found in southern England and Wales, and north‐eastern and central Scotland (Figure 2G). The uncertainty of our predictions was generally low, with some small hotspots of higher uncertainty (Figure 3). Additionally, the predictions for several species—particularly *B. pendula* , *F. sylvatica* , *F. excelsior* , *Q. robur* and *P. sylvestris* —exhibited higher uncertainty in Scotland (Figure 3B–EI).

When we examined the sensitivity of our projections to the resolution of the spatial mesh (Table S2, Figures S1–S4), we found that the results for *A. pseudoplatanus* were similar regardless of the choice of mesh. Furthermore, the results for *B. pendula* , *F. sylvatica* and *S. aucuparia* were similar for all but the coarsest mesh. For *P. sitchensis* , the predictions were similar for the 5, 10 and 20 km meshes; the 30 km predictions exhibited many of the same qualitative patterns, but with higher predicted intensity in individual grid cells in Scotland. *F. excelsior* displayed more pronounced hotspots in the finest mesh and some qualitative differences in the coarsest mesh; however, the major hotspot in East Anglia persisted across all five meshes. *P. sylvestris* also exhibited more pronounced hotspots in the finest mesh, but the general pattern of higher predicted occurrence in Scotland persisted across all choices of mesh. The results which were most sensitive to mesh choice were those for *P. abies* and *Q. robur* .

**FIGURE 4:**
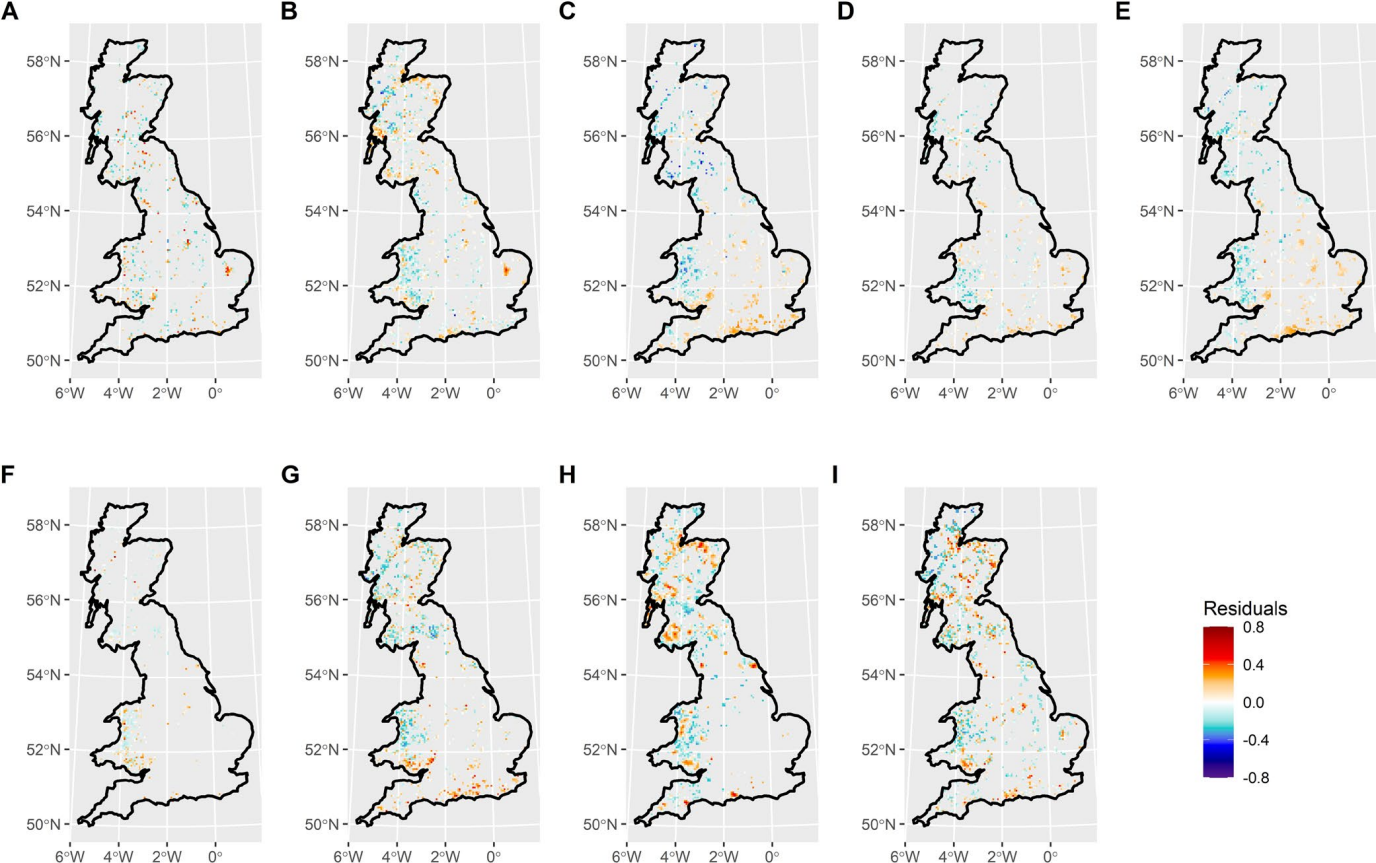
Residuals of pest and disease intensity modelled as a function of host tree species cover for (A) *Acer pseudoplatanus* , (B) *Betula pendula* , (C) *Fagus sylvatica* , (D) *Fraxinus excelsior* , (E) *Quercus robur* , (F) *Sorbus aucuparia* , (G) *Picea abies* , (H) *Picea sitchensis* and (I) *Pinus sylvestris* . Positive values (red) indicate areas where pest and disease intensity is higher than expected for the level of host tree cover, while negative values (blue) show areas where intensity is lower than expected. Map lines delineate study areas and do not necessarily depict accepted national boundaries.

Predicted pest and disease intensity for *F. sylvatica* and *Q. robur*, and to a lesser extent *F. excelsior*, was generally higher than expected for the level of host tree cover in the south and east of England and lower than expected in Scotland and Wales (Figures 4C,E). A similar pattern was also observed for one conifer, *P. abies* (Figure 4G). By contrast, the other conifers— *P. sitchensis* and *P. sylvestris* —had higher than expected intensity in much of Scotland, as well as parts of England and Wales (Figure 4H,I). *B. pendula* also exhibited higher than expected intensity in parts of Scotland, but generally lower than expected intensity in Wales (Figure 4B). *A. pseudoplatanus* did not exhibit clear patterns, but did have several hotspots where intensity was higher than expected (Figure 4A). *S. aucuparia* was represented as the primary, secondary or tertiary species in relatively few sub‐compartments outside of Wales and south‐west Scotland. However, in Wales the pest and disease intensity for this host appeared to be generally higher than expected in the south, but lower in the north (Figure 4F).

### 3.2 Drivers of Pest and Disease Occurrence

**FIGURE 5:**
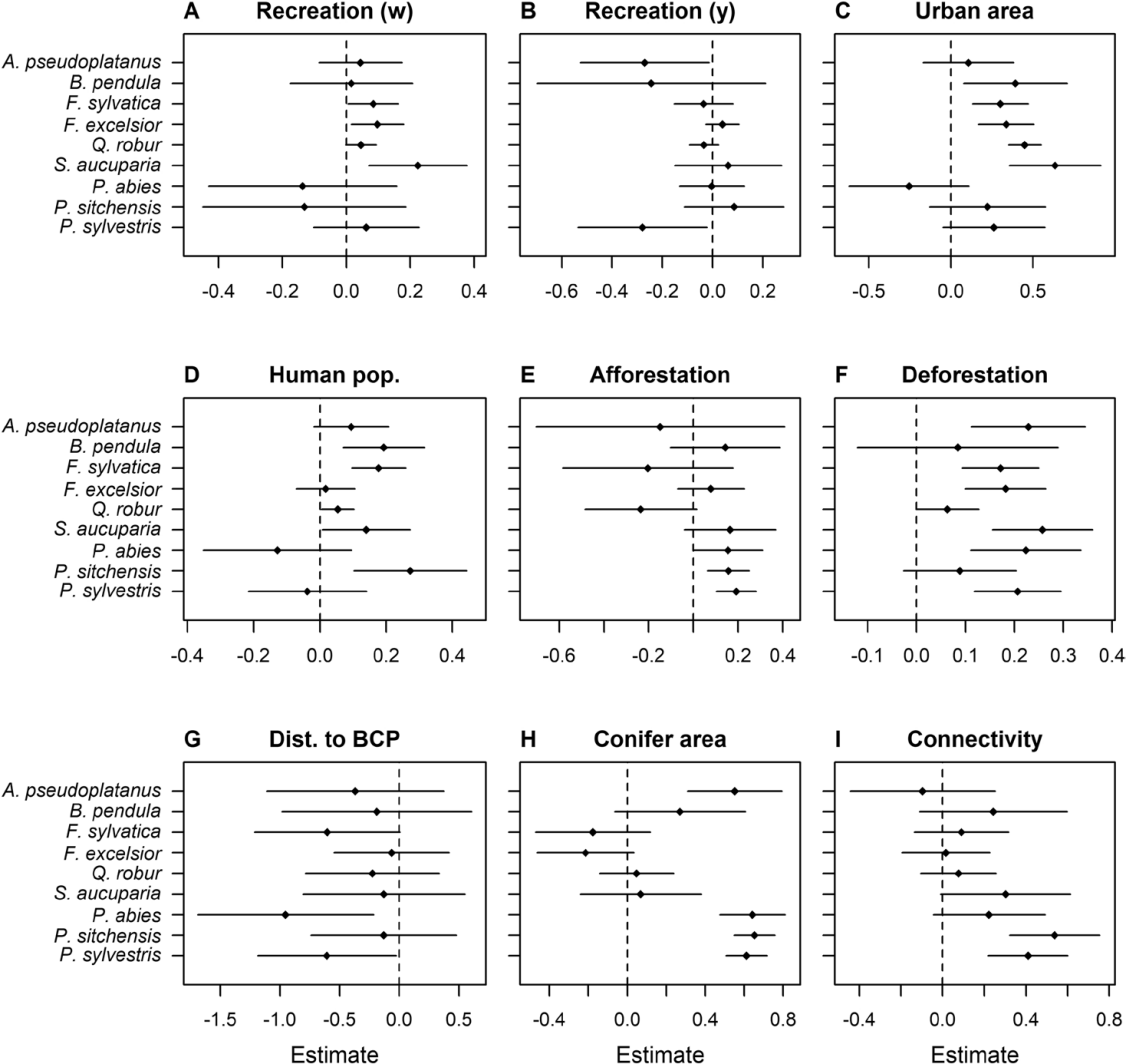
Posterior mean effect (±95% compatibility interval) of nine focal variables on pest and disease occurrence for nine host tree species. Effect estimates were produced by ISDMs using a maximum edge length of 5 km. Dist. to BCP = distance to border control post. Recreation (w) = weekly recreation, recreation (y) = yearly recreation.

Based on the assumptions embodied in our maximal DAG (Figure 1), the effects of our nine focal variables on pest and disease occurrence varied among the host tree species (Figure 5). Weekly recreation had a relatively strong positive effect on pest and disease occurrence for *S. aucuparia* and weaker positive effects for *F. sylvatica, F. excelsior* and *Q. robur* (Figure 5A). By contrast, yearly recreation did not have positive effects for any host species and had negative effects for *A. pseudoplatanus* and *P. sylvestris* (Figure 5B). Urban area had positive effects, increasing pest and disease occurrence for five of the host species, all broadleaves (Figure 5C). Furthermore, the majority of the posterior probability for two of the conifer species— *P. sitchensis* and *P. sylvestris* —was also positive, but the 95% compatibility intervals (i.e., credible intervals) did overlap zero for both species (Figure 5C). Human population also had clear positive effects on pest and disease occurrence for *B. pendula, F. sylvatica* and *P. sitchensis* ; there was also some evidence for positive effects for *A. pseudoplatanus* , *Q. robur* and *S. aucuparia* , although for these species values around zero were also compatible with the model and the data (Figure 5D). Afforestation and deforestation were both positively related to pest and disease occurrence for several of the host tree species (Figures 5E,F), with the latter variable having clear positive effects for all but three hosts. Distance to border control post did not have clear effects for most of the host tree species, but greater distances were associated with lower pest and disease occurrence for *P. abies* , and to a lesser extent *P. sylvestris* and *F. sylvatica* (Figure 5G). Conifer coverage had clear positive effects on pest and disease occurrence for the three conifer host species and also for one broadleaf— *A. pseudoplatanus* (Figure 5H). Finally, woodland connectivity had clear positive effects on pest and disease occurrence for two host species— *P. sitchensis* and *P. sylvestris* (Figure 5I). The majority of the posterior probability for the effect was also positive for *P. abies* and *S. aucuparia* , but the 95% compatibility intervals did cover effects around zero for both species.

Our inferences were generally insensitive to varying the assumptions embodied in our DAG (Figure 1). We examined a total of 146 alternative DAG structures, between 13 and 28 for each focal variable (Figures S9–S16), which resulted in effect estimates from 67 models (Figures S17–S25) corresponding to minimum adjustment sets different to those implied by Figure 1. For afforestation and deforestation, all alternative DAGs implied the same model as Figure 1. For the other focal variables, in which at least one alternative model structure was implied, we obtained effect estimates that were generally similar to those that we present in Figure 5. One exception was the effect of weekly recreation for *S. aucuparia* , where one model produced a strong negative effect, and another produced an effect estimate centred on zero (Figure S17). However, as the other 21 alternative models all produced qualitatively similar estimates to the effect presented in Figure 5, we argue that our main estimate of this effect is relatively robust to our modelling assumptions. A second notable exception was the negative effect of yearly recreation for *A. pseudoplatanus* ; although the posterior mean effect was negative for all model variants, the 95% compatibility intervals substantially overlapped zero for all but one of the 23 alternative models (Figure S18). Therefore, we cannot be confident that yearly recreation has a negative effect on pest and disease occurrence for *A. pseudoplatanus* .

The effects of some of the focal drivers were sensitive to the resolution of the spatial mesh (Figure S26). The main qualitative differences in the results for the coarse mesh, relative to those for the finely grained mesh, were that several species exhibited strong negative effects of weekly recreation, while yearly recreation had clear positive effects for several species. Furthermore, the effect of urban area for *Q. robur* was very strongly positive, while *P. sitchensis* and *P. sylvestris* exhibited strong negative relationships with distance to border control post. Conifer area had very strong positive effects for several species, and the effects of connectivity were more strongly positive for the coarse mesh than the results for the fine mesh.

## 4 Discussion

Our analysis of 18,871 pest and disease records covering 22 years in mainland Great Britain shows that the spatial distribution of tree pest and disease burdens in the UK varies among nine nationally significant host tree species. Predicted pest and disease intensity for several of the broadleaved hosts exhibited hotspots of high intensity in England, while predictions for conifers were high in much of Scotland. Urban area, human population density, and weekly (i.e., relatively local) recreation were important drivers for several host species, mainly native broadleaves. For conifer hosts, woodland connectivity, afforestation and conifer coverage were the main drivers of pest and disease burdens. Deforestation was also an important driver for both conifer and broadleaf hosts. These findings have implications for the management of pest and disease threats to the UK's treescapes.

Several of the broadleaved species in our sample— *A. pseudoplatanus* , *F. sylvatica, F. excelsior, Q. robur* and *S. aucuparia* —exhibited patches of high predicted pest and disease intensity in England. In some cases, these hotspots appeared to coincide with London and other major cities, which is consistent with our finding that urban area and human population were drivers of pest and disease intensity for several hosts, as discussed below. Other hotspots may be a result of historic outbreak dynamics; for instance, the high predicted intensity for *F. excelsior* in East Anglia is likely due to this area being the initial site of the ash dieback (*Hymenoscyphus fraxineus*) outbreak in the UK (Potter and Urquhart 2017). The predicted intensity for two of the conifer species— *Picea sitchensis* and *Pinus sylvestris* —also displayed hotspots in England and Wales, which may reflect the fact that these species are not native to the region (Hall et al. 2004) and are thus mainly present in commercial plantations (Forestry Commission 2014, 46). By contrast, larger areas of high intensity were predicted for Scotland. This likely reflects the increased prevalence of conifer forests (Forest Research 2024, 8), which, for *P. sylvestris* , include native pinewoods (Salmela et al. 2010). As the host tree species in our sample represent some of the most widespread and abundant species in UK forests, our findings are broadly informative of the overall distribution of tree pest and disease risk in the UK. Given the high levels of robustness to varying modelling assumptions, alongside the biologically interpretable driver effects discussed below, our models could be used to guide the allocation of resources for surveillance and management for the modelled host species, though further engagement with stakeholders is needed to understand how these could be tailored to decision‐making. Resource allocation decisions would also need to consider other factors including the cost of surveying different regions, the qualities of different surveillance tools, the expected damage costs for different pests and the effectiveness of different management options (Epanchin‐Niell et al. 2012, 2014; Nguyen et al. 2024; Roberts et al. 2020). Future research could complement our study by producing predictions for individual pest and disease species, particularly high‐risk species such as *Phytophthora ramorum*. Furthermore, our predictions could be integrated with other data, such as the distribution of environmental stressors other than pests and diseases, to gain a more holistic view of the threat to UK treescapes. An especially important topic for future research is the effect of climate change on pest and disease burdens in UK treescapes; predicting future pest and disease burdens will require consideration of factors such as the emergence of new pests and diseases, potential shifts in the distributions of primary or secondary host species and comparison of different emissions scenarios.

We found that urban area and human population were clear drivers of pest and disease intensity for several host species. These effects are consistent with empirical evidence from other systems. For example, Colunga‐Garcia et al. (2010) found that the species richness of 39 priority tree pests and diseases in the contiguous United States followed a clear urban–rural gradient, with higher richness in more urbanised areas. Similarly, first detection locations for non‐native insect pests in Europe are strongly associated with distance to the nearest city (Branco et al. 2019). Several processes may contribute to these effects, including the prevalence of non‐native host trees (Branco et al. 2019), the risk of propagules being transported on live plants and subsequently introduced to gardens (Paap et al. 2017) and reduced abundance of arthropod predators which would otherwise help to control insect pests (Korányi et al. 2022). Physiological stress owing to factors including pollution, soil compaction and pruning can also leave host trees more vulnerable to pests and diseases (Pautasso et al. 2015). In addition, urban areas can pose logistical challenges which hinder the effective management of pest outbreaks (Tomlinson et al. 2015). In principle, detectability of pests could be higher for urban trees because more people are present to spot an affected tree. However, we do not believe that detectability can account for the observed effects because the majority of data for host trees with a positive effect of urban area came from the APHA dataset (Table 1), which is derived from visits from plant health inspectors (by contrast, the THDAS dataset comprises public reports) and also accounts for survey effort by recording the number of visits to each location. Furthermore, the positive effects of urban area were robust to the inclusion of human population density (Figures S10 and S19), which is positively related to the probability that a pest is detected by the public rather than other sources (Epanchin‐Niell and Pi 2024). Given the damage that pests and diseases can cause to urban trees and the ecosystem services they provide (Raum et al. 2023), our results highlight urban treescapes as key targets for surveillance and management efforts.

We also found that weekly recreation—but not yearly recreation—was a driver of pest and disease burdens for several host species. We used recreation data which comprise the predicted number of non‐vehicular recreational visits (e.g., hiking, cycling) to an area, for a given frequency of activity. Assuming that weekly recreation captures information on relatively local (e.g., daily or weekend) visits to an area, while the yearly data represents longer distance and/or duration (e.g., summer holiday) visits, our findings suggest that activities which occur on a relatively frequent and local scale may contribute more to total pest and disease burdens than less frequent long‐distance excursions. There are several potential explanations for this result. For instance, it may be that there are simply many more local recreational visits than long‐range visits and hence more chances for pests and pathogens to be transported and introduced. There may also be differences in propagule survival between frequently and infrequently used footwear and equipment, or differences in cleaning behaviour prior to engaging in local versus long‐distance activities, but to our knowledge these effects have not been investigated in the context of tree pests and diseases in the UK. We suggest that a more detailed investigation into the role of short‐distance recreation in pest and disease spread would provide a more detailed understanding of how recreation affects pest and disease spread and could inform more effective mitigation strategies. For instance, if activities which incur particularly high risk of transporting pests and diseases were identified, then they could be targeted by tailored biosecurity awareness campaigns (e.g., the Scottish Forestry ‘Keep it Clean’ campaign; Scottish Forestry 2025). Similar campaigns have been used to target specific recreational activities, such as fishing and water sports, which have a high risk of introducing freshwater invasive species (Anderson et al. 2015; Chapman et al. 2020). We emphasise that our findings do not suggest that long‐distance recreation‐mediated dispersal is unimportant; long‐distance dispersal events could still allow pests and diseases to invade new regions, enabling subsequent dispersal by local recreation and other vectors. Long‐distance dispersal can also substantially reduce the probability of successfully containing an outbreak by quarantining affected areas (Strona et al. 2020). Finally, differences between non‐native and native pests and pathogens may influence the relative importance of short versus long‐distance dispersal. For instance, evidence from North America suggests that non‐native insect pests tend to be associated with a broader range of hosts than native pests (Wang et al. 2022), which may increase the probability of successful long‐distance dispersal by increasing the probability that a suitable host is available. Consequently, long‐distance dispersal may be more important for the spread of non‐native than native insect pests—future research could aim to test this prediction in UK treescapes.

Woodland connectivity was positively related to pest and disease intensity for two conifers, *P. sitchensis* and *P. sylvestris* . The importance of connectivity in pest and disease spread has been highlighted in previous research; for instance, connectivity is thought to have been a key determinant of pine wilt disease spread in China (Huang et al. 2024) and to have played a role in the spread of *Phytophthora ramorum* in the western United States (Ellis et al. 2010) and Great Britain (Purse et al. 2016). However, increasing woodland connectivity can also be important for forest conservation; across a range of plant and animal taxa, improving connectivity generally results in increased species occurrence and diversity (Humphrey et al. 2015). Notably, increasing forest diversity can confer resilience against pest and disease outbreaks (Field et al. 2025). As *P. sitchensis* and *P. sylvestris* are plantation species in the UK (except for Scotland, where native *P. sylvestris* accounts for around 15% of pinewood area; Salmela et al. 2010) and historically were usually planted in monoculture (Kerr 1999), the fact that only these two species showed a clear positive effect of connectivity may be explained if they received the cost of increased connectivity (i.e., increased propagule pressure from pests and pathogens) without the protective effect of increased biodiversity. We suggest that exploring the interplay and potential trade‐offs between woodland connectivity, biodiversity, and pest and disease outbreaks is an important topic for future research.

Afforestation and deforestation were associated with increased pest and disease burdens for several tree species. Forestry activity may lead to increased pest and disease burdens due to the introduction of propagules on equipment and machinery (Jules et al. 2002; Riddell et al. 2024). Furthermore, propagules can be introduced on live plants during restocking and restoration; our findings are consistent with evidence highlighting the potential risks of introducing pathogens via planting and the need for improved biosecurity in supply chains (Donald et al. 2021; Dunn et al. 2021; Green et al. 2025; Sims and Garbelotto 2021). Additionally, the effect of afforestation may include legacy effects of agricultural land which is converted to forest (Stritih et al. 2021); establishing how agricultural legacy effects influence pest and disease outbreaks in the UK would be a productive avenue for future research. However, we suggest that our results regarding afforestation and deforestation should be interpreted with some caution. Although we accounted for sampling effort through the inclusion of presence‐absence data and followed current best practices by including a second spatial field for our presence‐background data (Simmonds et al. 2020), we cannot completely exclude the possibility that our estimated effects have been inflated due to differences in the ability of different forest users to identify pests. For example, experienced foresters may be more able than other forest users to accurately distinguish between symptoms which indicate a pest and symptoms which are not concerning. Foresters may thus be less likely to submit reports to APHA which lead to a survey but no pest detection. This would reduce the number of absences relative to presences, thus making pest and disease burdens appear higher in areas where experienced foresters are operating. However, as Forest Research's Tree Alert tool (which feeds into the presence‐background THDAS data) is the primary avenue for reporting issues with trees, the magnitude of this effect may be relatively small. Overall, although we cannot be certain that the effects of afforestation and deforestation on pest and disease intensity are not due to sampling effort, we believe that investigating whether forestry activities lead to pest and disease spread in the UK is an important avenue for future research.

Our study highlights the strength of integrated species distribution models (ISDMs) in a real‐world application; by combining two different sources of pest and disease data, we were able to exploit the spatial coverage of a presence‐background dataset arising from opportunistic public reports, while also leveraging the information on sampling effort contained within the presence‐absence plant health monitoring data. Furthermore, we did not encounter issues with the spatial random effects which have been reported in previous work. Specifically, as we increased the resolution of our spatial mesh from 40 km to 5 km, we did not observe patterns of overfitting—in which the predicted intensity is strongly clustered around the observed datapoints—as observed by Dambly et al. (2023). We suggest that our penalised complexity prior more strongly penalised large values of the standard deviation for the Matérn covariance function (*p*(*σ* > 1) = 0.05, c.f. *p*(*σ* > 1) = 0.5 in Dambly et al. 2023), limiting the tendency of our spatial random field to exhibit large values around the observed datapoints. In addition, we used regularising priors for the fixed effects (precision = 1, c.f. precision = 0.001 in Dambly et al. 2023); as priors are defined on the linear predictor scale, but intensity equals the exponential function of the linear predictor, specifying a flat prior for the fixed effects places a large amount of prior probability at extremely large and small intensity values. This may exacerbate overfitting where covariates are involved.

We have shown that the total pest and disease burdens for a set of nationally significant host tree species in the UK are spatially variable, with some species exhibiting pronounced hotspots which could be targeted for further surveillance and management to improve early detection of pest and disease outbreaks when eradication or containment are still feasible. The drivers of pest and disease intensity also varied among hosts, but urban area, human population density and recreation were important for several species, mainly native broadleaves. Woodland connectivity was also important for two conifer species. We also found that afforestation and deforestation were also associated with increased pest and disease burdens for several hosts, although we note that these effects should be interpreted with caution due to the potential effects of sampling effort. We also note that as the UK has relatively low tree cover (10% in England, 19% in Scotland and 15% in Wales; Office for National Statistics 2024), caution should be exercised when applying our findings to more heavily forested countries. Our findings have implications for the management of forests to combat the ongoing threat posed by tree pests and diseases. Furthermore, our findings also highlight the importance of integrating landscape cover and connectivity metrics, reflecting different pathways of propagule spread, into predictive models of pest and disease patterns. Finally, our study illustrates the strength of using integrated species distribution models to combine disparate data on species occurrence.

## Author Contributions

**Peter S. Stewart:** conceptualization, data curation, formal analysis, investigation, methodology, software, visualization, writing – original draft, writing – review and editing. **Louise J. Barwell:** conceptualization, data curation, investigation, software, writing – review and editing. **Katharine Turvey:** data curation, investigation, software, writing – review and editing. **Jane Barbrook:** data curation, writing – review and editing. **Sarah Green:** data curation, writing – review and editing. **Ana Pérez‐Sierra:** data curation, writing – review and editing. **Bethan V. Purse:** conceptualization, data curation, funding acquisition, software, supervision, writing – review and editing. **Daniel Chapman:** conceptualization, data curation, funding acquisition, investigation, software, supervision, writing – review and editing.

## Funding

This work was supported by the Natural Environment Research Council (NE/V020005/1).

## Conflicts of Interest

The authors declare no conflicts of interest.

## Supporting information


**Data S1:** gcb70706‐sup‐0001‐Supinfo.pdf.

## Data Availability

Code to fully reproduce our analyses is available on Zenodo at: https://doi.org/10.5281/zenodo.15641235. The pest and disease data provided by Forest Research and the Animal and Plant Health Agency (APHA) are not publicly available under the terms of the original data‐sharing agreements. UKCEH land cover and land cover change data are available from https://doi.org/10.5285/35c7d0e5‐1121‐4381‐9940‐75f7673c98f7 and https://doi.org/10.5285/07b6e5e9‐b766‐48e5‐a28c‐5b3e35abecc0, respectively. Gridded UK population data are available from https://doi.org/10.5285/0995e94d‐6d42‐40c1‐8ed4‐5090d82471e1. OS Open Roads data are available from https://osdatahub.os.uk/downloads/open/OpenRoads. Vascular plant α‐diversity data are available from https://doi.org/10.25829/idiv.3506‐p4c0mo. BCP location data are available from https://planthealthportal.defra.gov.uk/trade/imports/imports‐from‐the‐eu/bcpscps/bcp‐locations‐and‐map/. HydroSHEDS and HydroRIVERS data are available at https://www.hydrosheds.org/hydrosheds‐core‐downloads and https://www.hydrosheds.org/products/hydrorivers, respectively. National Forest Inventory data are available from https://www.forestresearch.gov.uk/tools‐and‐resources/fthr/open‐data/. Recreation demand data are available from https://doi.org/10.5285/bd3bf607‐a3b2‐423b‐b07b‐9c41e84746ee. Ancient Woodland Inventory data are available from https://naturalengland‐defra.opendata.arcgis.com/datasets/Defra::ancient‐woodland‐england/about, https://datamap.gov.wales/layers/inspire‐nrw:NRW_ANCIENT_WOODLAND_INVENTORY_2021 and https://opendata.nature.scot/datasets/ancient‐woodland‐inventory/explore. Registered park and garden data are available from https://portal.historicenvironment.scot/apex/f?p=PORTAL:downloads:::::DATASET:ALL, https://opendata‐historicengland.hub.arcgis.com/datasets/historicengland::national‐heritage‐list‐for‐england‐nhle/about?layer=7 and https://datamap.gov.wales/layergroups/geonode:registered_historic_parks_and_gardens. Elevation data are available from https://sdi.eea.europa.eu/catalogue/srv/api/records/3473589f‐0854‐4601‐919e‐2e7dd172ff50. Vapour pressure deficit data are available from https://doi.org/10.5285/bbca3267dc7d4219af484976734c9527. Forest sub‐compartment data are available from: https://data‐forestry.opendata.arcgis.com/datasets/0750654e96e34832ab24554ba35bfb5d_0/about and https://datamap.gov.wales/layers/inspire‐nrw:NRW_PRODUCTISED_SCDB_LLE.
