# Supplementary material for: Patterns and Drivers of Pest and Disease Occurrence in UK Treescapes
Source: Glob Chang Biol. 2026 Feb 6;32(2):e70706. doi: 10.1111/gcb.70706 (PMC12881709; doi:10.1111/gcb.70706)
Supplement: Supplementary file 1 — Data S1: gcb70706‐sup‐0001‐Supinfo.pdf. [file GCB-32-e70706-s001.pdf]

Supplementary material: Patterns and drivers of pest and disease occurrence in UK treescapes

Peter S. Stewart[1,2]* Louise J. Barwell[3], Katharine Turvey[3], Jane Barbrook[4], Sarah Green[5], Ana Pérez-Sierra[6], Bethan V. Purse[3], Daniel Chapman[2]

1. School of Mathematics and Statistics, University of Glasgow, Glasgow, UK.

2. Biological and Environmental Sciences, University of Stirling, Stirling, UK.

3. UK Centre for Ecology and Hydrology, Wallingford, UK.

4. Animal and Plant Health Agency, Plant Health and Seeds Inspectorate, Sand Hutton, York YO41 1LZ, UK

5. Forest Research, Northern Research Station, Roslin, UK.

6. Forest Research, Alice Holt Lodge, Farnham, UK.

***Corresponding author address:** peter.s.stewart@glasgow.ac.uk. School of Mathematics and Statistics, University of Glasgow, Glasgow, UK. G12 8QQ.

Supplementary methods

Selection of tree genera for APHA data

The 19 priority tree genera were selected from 70 species on the Silvifuture list (https://www.silvifuture.org.uk/species). The list was reduced to 37 species within 19 genera through consultation with experts in silviculture and forest health to identify and exclude species unsuitable for UK use because of poor growth or severe pest or disease impacts.

Explanation of causal assumptions

We used directed acyclic graphs (DAGs) to represent our assumptions about the mechanisms which may underly pest and disease occurrence. Importantly, DAGs are non-parametric in the sense that they do not require any assumptions about the specific shape of the functional relationships between variables (Pearl, 1995).

In our maximal directed acyclic graph (DAG; Figure 1 main text) we assumed that pest and disease occurrence (y) is a function of the probability that a pest is introduced to an area (p_i) and the probability that a pest subsequently establishes (p_i). Furthermore, we assumed that establishment probability is influenced by the probability of introduction, as establishment is known to be influenced by propagule pressure (Simberloff, 2009). We assumed that both probabilities are latent (*i.e.,* unobserved). We further assumed that a variety of potential drivers could directly or indirectly influence either introduction, establishment, or both. In our analysis, we focused on eight drivers: recreation, urban area, human population, afforestation, deforestation, distance to the nearest border control point, conifer area, and woodland connectivity.

Recreation could influence introduction due to the potential for people to transport propagules, for instance on footwear, clothing, or sporting equipment. This is supported by evidence from other systems; for instance, Cushman and Meentemeyer (2008) found that *Phyotphthora ramorum* is more prevalent in soil samples taken from hiking trails than in samples taken from nearby non-trail areas. Furthermore, they observed that at a landscape scale, areas accessible to recreationists had more disease than non-accessible areas (Cushman and Meentemeyer, 2008). In the UK, dispersal by recreationists is considered to be a potential pathway for pest and pathogen spread (*e.g.,* Hall *et al.*, 2019).

Human population is assumed to affect recreation, as people are more likely to perform common types of 'day-to-day' recreation (*e.g.,* dog walking, jogging) close to where they live (Graham and Eigenbrod, 2019). Additionally, human population could directly influence introduction, for instance due to the movement of propagules (*e.g.,* on footwear or vehicles) during non-recreational activities.

There is clear evidence from other systems that pest and disease prevalence often follows an urban rural gradient (Branco *et al.*, 2019; Colunga-Garcia *et al.*, 2010). We assumed that urban area is assumed to influence introduction, because pest and pathogen propagules can be introduced during planting in private gardens (Paap *et al.*, 2017). We also assumed that urban area can influence establishment, for instance by increasing non-native host availability (Branco *et al.*, 2019), increasing host physiological stress (Pautasso *et al.*, 2015), decreasing biotic resistance (Korányi *et al.*, 2022), and hampering management efforts (Tomlinson *et al.*, 2015). We further assumed that urban area is a function of human population, due to the link between population growth and urban land expansion (Mahtta *et al.*, 2022). Finally, we assumed that urban area may affect recreation (in addition to any indirect effect of human population), because the tendency for people to visit an area could be influenced by how built-up it is.

Afforestation and deforestation are assumed to directly influence the probability of introduction, which is supported by evidence that propagules can be introduced on forestry machinery (Jules *et al.*, 2002). In addition, propagules can be introduced on live plants during afforestation (Donald *et al.*, 2021; Dunn *et al.*, 2021). We also assumed that both afforestation and deforestation can influence woodland, broadleaf, and conifer area, as large areas of trees may be planted or felled. Additionally, we assumed that ancient woodland area may be affected by deforestation, but not afforestation, as ancient woodlands could have been removed (but not planted) between 1990 and 2015.

Border control posts (BCPs) are sites where plants and plant products entering the UK are inspected (DEFRA, 2025). We assumed that introduction could be influenced by the distance to the nearest BCP, due to the potential for propagules on imported produce to escape into the wider environment. Distance to border control point is assumed to be influenced by elevation and urban area, as most border control points are coastal and close to relatively built-up areas.

Conifer area is assumed to influence introduction and establishment, for instance by influencing the availability of suitable hosts for conifer pests and diseases. Additionally, as the majority of conifer forest in the UK is plantations (Salmela *et al.*, 2010), conifer area may also act as a proxy of forestry activity. As discussed above, forestry activity may lead to propagules being introduced on machinery and planted trees (Donald *et al.*, 2021; Dunn *et al.*, 2021; Jules *et al.*, 2002). We also assumed that conifer area can affect recreation, as both natural and plantation conifer forests can provide suitable locations for outdoor recreation (Hahn *et al.*, 2010). We assumed that conifer area is affected by urban area, as the two cover types are mutually exclusive.

Woodland connectivity has been shown to influence the dynamics of pest and disease spread, facilitating the dispersal of pests and diseases to new locations (Ellis *et al.*, 2010; Huang *et al.*, 2024; Purse *et al.*, 2016). Consequently, we assumed that woodland connectivity can affect the probability of introduction. As our woodland connectivity metric is derived from the woodland area layer (see main text), we assumed that connectivity is a function of woodland area. We also assumed that recreation could be influenced by woodland connectivity, for instance by affecting the desirability or accessibility of different areas.

In addition to our eight focal drivers, we considered a further six variables which could potentially mediate and/or confound the focal drivers' effects. These variables were: vapour pressure deficit (VPD), elevation, woodland area, broadleaf area, ancient woodland area, and distance to park/garden.

VPD is defined as the difference between level of water vapour in the air, and the level of water vapour at which the air is saturated (Novick *et al.*, 2024). It is a function of temperature and humidity (Novick *et al.*, 2024), which influence outbreak dynamics by affecting microclimate suitability for pests and diseases and by causing physiological stress for host trees (Mosedale *et al.*, 2024; Novick *et al.*, 2024; Romero *et al.*, 2022). VPD is related to elevation (Gotsch *et al.*, 2017), and can be affected by urbanisation (Gong *et al.*, 2024).

In addition to its effect on VPD, elevation potentially influences a number of the other variables which we consider. As mentioned above, BCPs are generally found in low-lying coastal areas. We further assumed that urban area and human population are both influenced by elevation, as high-elevation areas tend to be unsuitable for building and are often sparsely populated (Mansfield, 2017). We also assumed that elevation can affect recreation, as upland areas are often popular for recreational activities such as hiking (Gilchrist *et al.*, 2023). Finally, we assumed that woodland area, broadleaf area, and conifer area are affected by elevation due to the presence of the tree line in UK upland areas (Grace, 1997).

Broadleaf area and woodland area, like conifer area, are assumed to influence introduction and establishment; these effects may operate via a multitude of pathways, such as via affecting host availability and biotic resistance (Gougherty and Davies, 2024). Furthermore, like conifer area, we

assumed that broadleaf and woodland area can affect recreation (Graham and Eigenbrod, 2019). We also assumed that both woodland and broadleaf area are affected by urban area, as these land types are mutually exclusive. In addition, we assumed that woodland area is a function of both conifer and broadleaf area, as they comprise a subset of woodlands.

Ancient woodland area is assumed to influence woodland area, as ancient woodlands are also a subset of woodlands. Additionally, ancient woodland area is assumed to influence recreation, as ancient woodlands may be attractive locations for people to visit. We assumed that ancient woodland area is influenced by urban area, as the two cover types are mutually exclusive. There is also the potential for fragmentation of ancient woodland area due to nearby development (Corney *et al.*, 2008).

Finally, distance to park/garden is assumed to influence recreation, as people may travel to visit these areas and use them for walking or other activities. Distance to park/garden may also influence introduction and establishment, for example due to the high prevalence of non-native plants which may act as primary or secondary hosts, the risk of pests being introduced during ornamental planting, and the potential for gardens and horticulture to act as sources for pests and diseases to spill over into surrounding areas (Barham *et al.*, 2016; Potter and Urquhart, 2017; Wondafrash *et al.*, 2021).

References

Barham, E., Sharrock, S., Lane, C., Baker, R., 2016. The International Plant Sentinel Network: a tool for Regional and National Plant Protection Organizations. *EPPO Bulletin* 46, 156–162. https://doi.org/10.1111/epp.12283

Branco, M., Nunes, P., Roques, A., Fernandes, M.R., Orazio, C., Jactel, H., 2019. Urban trees facilitate the establishment of non-native forest insects. *NeoBiota* 52, 25–46. https://doi.org/10.3897/neobiota.52.36358

Colunga-Garcia, M., Magarey, R.A., Haack, R.A., Gage, S.H., Qi, J., 2010. Enhancing early detection of exotic pests in agricultural and forest ecosystems using an urban-gradient framework. *Ecological Applications* 20, 303–310. https://doi.org/10.1890/09-0193.1

Corney, P.M., Smithers, R., Garnett, B., Lush, M., Kirby, K., Peterken, G., Le Duc, M., Marrs, R., 2008. *The impacts of nearby development on the ecology of ancient woodland*. URL: https://www.woodlandtrust.org.uk/media/43620/impacts-of-nearby-development-on-the-ecology-of-ancient-woodland.pdf

Cushman, J.H., Meentemeyer, R.K., 2008. Multi-scale patterns of human activity and the incidence of an exotic forest pathogen. *Journal of Ecology* 96, 766–776. https://doi.org/10.1111/j.1365-2745.2008.01376.x

DEFRA, 2025. *UK Plant Health Information Portal: BCP and CP Overview*. URL https://planthealthportal.defra.gov.uk/trade/imports/imports-from-the-eu/bcpscps/bcp-and-cp-overview/

Donald, F., Purse, B.V., Green, S., 2021. Investigating the Role of Restoration Plantings in Introducing Disease—A Case Study Using Phytophthora. *Forests* 12, 764. https://doi.org/10.3390/f12060764

Dunn, M., Finger, A., Marzano, M., 2021. *Assessment of plant biosecurity risks to Scotland from large scale tree plantings for environmental benefits: project final report.* Scotland's Centre of Expertise for Plant Health (PHC).

Ellis, A.M., Václavík, T., Meentemeyer, R.K., 2010. When is connectivity important? A case study of the spatial pattern of sudden oak death. *Oikos* 119, 485–493. https://doi.org/10.1111/j.1600-0706.2009.17918.x

Gilchrist, A., Glentworth, J., Mohd Radzuan, H.S., Clay, G., 2023. *The Influence of Recreational Activity on Upland Ecosystems in the UK: A Review of Evidence*. Natural England.

Gong, S., She, D., Xia, J., Wang, T., 2024. Impacts of urbanization on urban-rural VPD disparities across different subregions in China: A quantitative analysis based on station-pairing and partial differential methods. *Atmospheric Research* 311, 107681. https://doi.org/10.1016/j.atmosres.2024.107681

Gotsch, S.G., Davidson, K., Murray, J.G., Duarte, V.J., Draguljić, D., 2017. Vapor pressure deficit predicts epiphyte abundance across an elevational gradient in a tropical montane region. *American Journal of Botany* 104, 1790–1801. https://doi.org/10.3732/ajb.1700247

Gougherty, A.V., Davies, T.J., 2024. Evolutionary history of host trees amplifies the dilution effect of biodiversity on forest pests. *PLOS Biology* 22, e3002473. https://doi.org/10.1371/journal.pbio.3002473

Grace, J., 1997. The oceanic tree-line and the limit for tree growth in Scotland. *Botanical Journal of Scotland* 49, 223–236. https://doi.org/10.1080/03746609708684868

Graham, L.J., Eigenbrod, F., 2019. Scale dependency in drivers of outdoor recreation in England. *People and Nature* 1, 406–416. https://doi.org/10.1002/pan3.10042

Hahn, K., Jensen, F.S., Koch, N.E., 2010. A review of forest recreation and human health in plantation forests. *Irish Forestry* 67

Hall, C., Marzano, M., O'Brien, L., 2019. *Biosecurity engagement with hikers: a study at Rowardennan, Scotland*. Report produced by Forest Research for Forestry Commission Scotland.

Huang, J., Zhao, X., Mo, X., Wu, J., Zhou, Y., Fang, G., 2024. Host vegetation connectivity is decisive for the natural spread of pine wilt disease. *Pest Management Science* 80, 5141–5156. https://doi.org/10.1002/ps.8240

Jules, E.S., Kauffman, M.J., Ritts, W.D., Carroll, A.L., 2002. Spread of an Invasive Pathogen Over a Variable Landscape: A Nonnative Root Rot on Port Orford Cedar. *Ecology* 83, 3167–3181. https://doi.org/10.1890/0012-9658(2002)083[3167:SOAIPO]2.0.CO;2

Korányi, D., Egerer, M., Rusch, A., Szabó, B., Batáry, P., 2022. Urbanization hampers biological control of insect pests: A global meta-analysis. *Science of The Total Environment* 834, 155396. https://doi.org/10.1016/j.scitotenv.2022.155396

Mahtta, R., Fragkias, M., Güneralp, B., Mahendra, A., Reba, M., Wentz, E.A., Seto, K.C., 2022. Urban land expansion: the role of population and economic growth for 300+ cities. *npj Urban Sustainability* 2, 1–11. https://doi.org/10.1038/s42949-022-00048-y

Mansfield, L., 2017. Upland resource management in Britain. *Geography* 102, 141–152. https://doi.org/10.1080/00167487.2017.12094024

Mosedale, J.R., Eyre, D., Korycinska, A., Everatt, M., Grant, S., Trew, B., Kaye, N., Hemming, D., Maclean, I.M.D., 2024. Mechanistic microclimate models and plant pest risk modelling. *Journal of Pest Science* 97, 1749–1766. https://doi.org/10.1007/s10340-024-01777-y

Novick, K.A., Ficklin, D.L., Grossiord, C., Konings, A.G., Martínez-Vilalta, J., Sadok, W., Trugman, A.T., Williams, A.P., Wright, A.J., Abatzoglou, J.T., Dannenberg, M.P., Gentine, P., Guan, K., Johnston, M.R., Lowman, L.E.L., Moore, D.J.P., McDowell, N.G., 2024. The impacts of rising vapour pressure deficit in natural and managed ecosystems. *Plant, Cell & Environment* 47, 3561–3589. https://doi.org/10.1111/pce.14846

Paap, T., Burgess, T.I., Wingfield, M.J., 2017. Urban trees: bridge-heads for forest pest invasions and sentinels for early detection. *Biological Invasions* 19, 3515–3526. https://doi.org/10.1007/s10530-017-1595-x

Pautasso, M., Schlegel, M., Holdenrieder, O., 2015. Forest Health in a Changing World. *Microbial Ecology* 69, 826–842. https://doi.org/10.1007/s00248-014-0545-8

Pearl, J., 1995. Causal Diagrams for Empirical Research. *Biometrika* 82, 669–688. https://doi.org/10.2307/2337329

Potter, C., Urquhart, J., 2017. Tree disease and pest epidemics in the Anthropocene: A review of the drivers, impacts and policy responses in the UK. *Forest Policy and Economics* 79, 61–68. https://doi.org/10.1016/j.forpol.2016.06.024

Purse, B.V., Schlenzig, A., Harris, C., Searle, K., 2016. *Risk of Phytophthora infection in woodland and larch fragments across Scotland.* https://doi.org/10.5285/29726cda-09f5-4661-8fd4-ddaa5555466a

Romero, F., Cazzato, S., Walder, F., Vogelgsang, S., Bender, S.F., van der Heijden, M.G.A., 2022. Humidity and high temperature are important for predicting fungal disease outbreaks worldwide. *New Phytologist* 234, 1553–1556. https://doi.org/10.1111/nph.17340

Salmela, M.J., Cavers, S., Wachowiak, W., Cottrell, J.E., Iason, G.R., Ennos, R.A., 2010. Understanding the evolution of native pinewoods in Scotland will benefit their future management and conservation. *Forestry: An International Journal of Forest Research* 83, 535–545. https://doi.org/10.1093/forestry/cpq036

Simberloff, D., 2009. The role of propagule pressure in biological invasions. *Annual Review of Ecology, Evolution, and Systematics* 40, 81–102. https://doi.org/10.1146/annurev.ecolsys.110308.120304

Tomlinson, I., Potter, C., Bayliss, H., 2015. Managing tree pests and diseases in urban settings: The case of Oak Processionary Moth in London, 2006–2012. *Urban Forestry & Urban Greening* 14, 286–292. https://doi.org/10.1016/j.ufug.2015.02.009

Wondafrash, M., Wingfield, M.J., Wilson, J.R.U., Hurley, B.P., Slippers, B., Paap, T., 2021. Botanical gardens as key resources and hazards for biosecurity. *Biodiversity Conservation* 30, 1929–1946. https://doi.org/10.1007/s10531-021-02180-0

Supplementary figures and tables

Table S1. Covariates used in the top ISDM model to predict pest and disease occurrence for nine host tree species.

Species	Covariates
Acer pseudoplatanus	Ancient woodland cover, broadleaf edge, recreation (yearly), urban area, vascular plant α-diversity, woodland area
Betula pendula	Ancient woodland cover, broadleaf area, broadleaf connectivity, broadleaf edge, canopy height, conifer area, conifer connectivity, deforestation, distance to park/garden, elevation, recreation (weekly), rivers, vascular plant α-diversity, woodland area, woodland connectivity
Fagus sylvatica	Conifer area, conifer connectivity, conifer edge, distance to border control post, Distance to park/garden, elevation, rivers, vascular plant α-diversity, VPD, woodland connectivity
Fraxinus excelsior	Afforestation, ancient woodland cover, broadleaf connectivity, canopy height, conifer edge, elevation, human population, Recreation (weekly), recreation (yearly), rivers, roads
Picea abies	Broadleaf connectivity, canopy height, conifer connectivity, conifer edge, deforestation, Distance to border control post, human population, recreation (yearly), rivers, roads, urban area, vascular plant α-diversity, woodland area
Picea sitchensis	Afforestation, distance to park/garden, Elevation, rivers, roads, woodland connectivity
Pinus sylvestris	Ancient woodland cover, conifer area, Conifer edge, deforestation, distance to border control post, distance to park/garden, elevation, human population, rivers, roads, vascular plant α-diversity, woodland edge
Quercus robur	Conifer area, Distance to park/garden, elevation, flow accumulation, human population, vascular plant α-diversity, VPD, woodland edge
Sorbus aucuparia	Ancient woodland cover, broadleaf edge, Deforestation, flow accumulation, recreation (weekly)

Table S2. Correlations between mean predicted pest and disease intensity when using different values for the maximum edge length of the spatial mesh. Correlations are calculated between each mesh choice and the finest mesh (5km maximum edge length). For spatial predictions from the 5km mesh, see main text Fig. 2. For spatial predictions from the other meshes, see supplementary figures S1-S4.

	Correlation with 5km mesh prediction			
**Species**	**10km**	**20km**	**30km**	**40km**
Acer pseudoplatanus	0.989	0.963	0.957	0.924
Betula pendula	0.983	0.967	0.960	0.516
Fagus sylvatica	0.995	0.984	0.967	0.511
Fraxinus excelsior	0.943	0.895	0.853	0.452
Quercus robur	0.726	0.533	0.157	0.124
Sorbus aucuparia	0.980	0.933	0.921	0.775
Picea abies	0.010	-0.053	0.713	0.275
Picea sitchensis	0.980	0.927	0.772	0.252
Pinus sylvestris	0.629	0.798	0.789	0.546

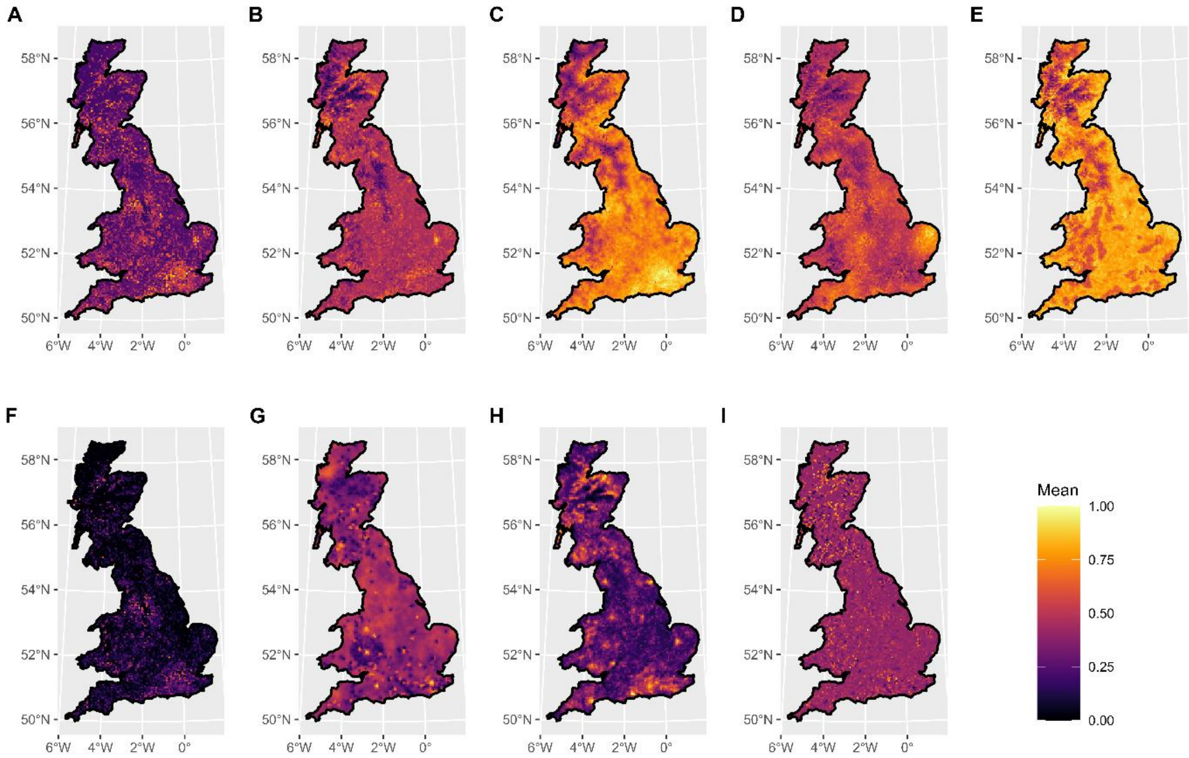

Figure S1. Predicted mean intensity of pest and disease occurrence, rescaled to a range of 0-1, for **A)** *Acer pseudoplatanus*, **B)** *Betula pendula*, **C)** *Fagus sylvatica*, **D)** *Fraxinus excelsior*, **E)** *Quercus robur*, **F)** *Sorbus aucuparia*, **G)** *Picea abies*, **H)** *Picea sitchensis*, and **I)** *Pinus sylvestris*. Predictions were generated from an ISDM using a maximum mesh edge length of 10km. For model covariates, see Table S1.

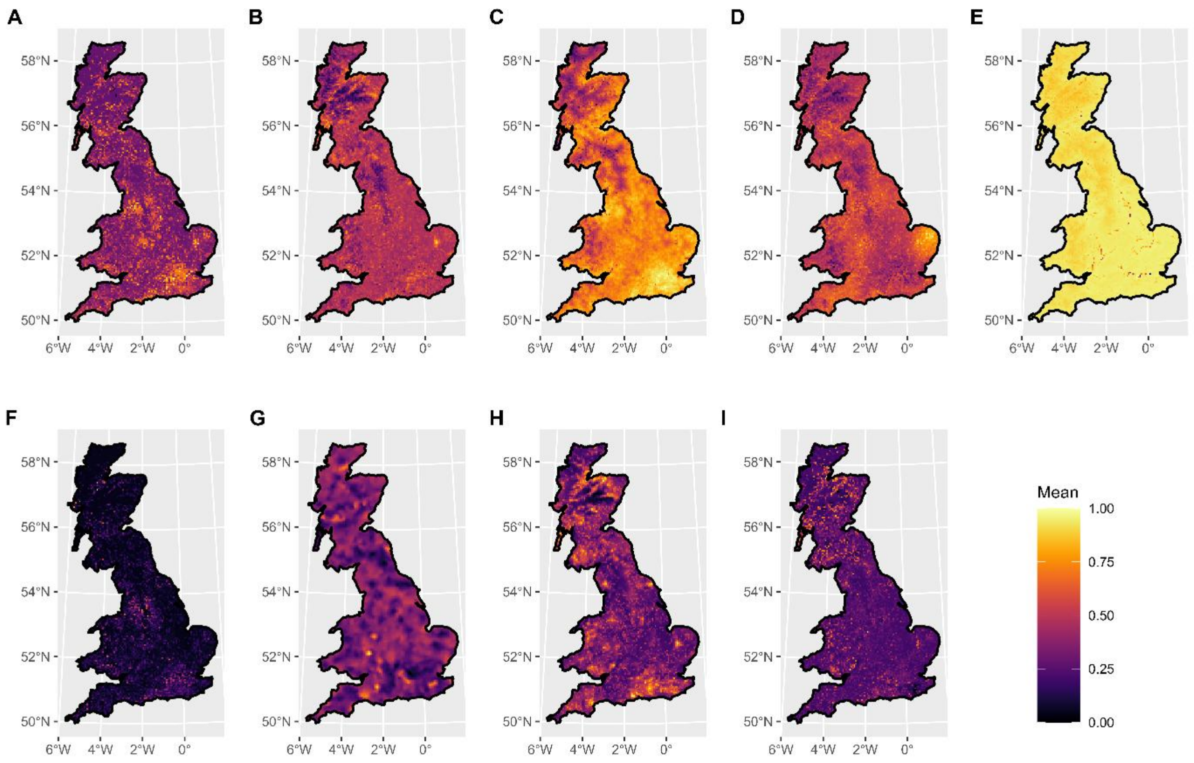

Figure S2. Predicted mean intensity of pest and disease occurrence, rescaled to a range of 0-1, for **A)** *Acer pseudoplatanus*, **B)** *Betula pendula*, **C)** *Fagus sylvatica*, **D)** *Fraxinus excelsior*, **E)** *Quercus robur*, **F)** *Sorbus aucuparia*, **G)** *Picea abies*, **H)** *Picea sitchensis*, and **I)** *Pinus sylvestris*. Predictions were generated from an ISDM using a maximum mesh edge length of 20km. For model covariates, see Table S1.

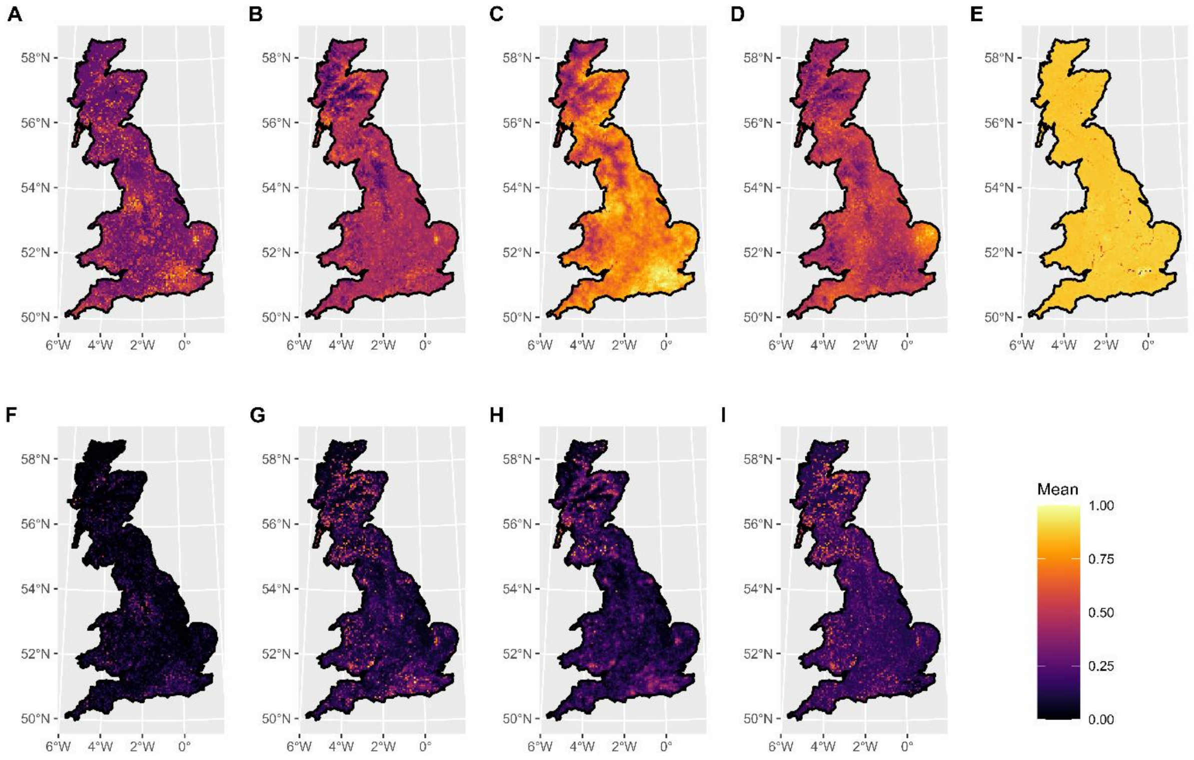

Figure S3. Predicted mean intensity of pest and disease occurrence, rescaled to a range of 0-1, for **A)** *Acer pseudoplatanus*, **B)** *Betula pendula*, **C)** *Fagus sylvatica*, **D)** *Fraxinus excelsior*, **E)** *Quercus robur*, **F)** *Sorbus aucuparia*, **G)** *Picea abies*, **H)** *Picea sitchensis*, and **I)** *Pinus sylvestris*. Predictions were generated from an ISDM using a maximum mesh edge length of 30km. For model covariates, see Table S1.

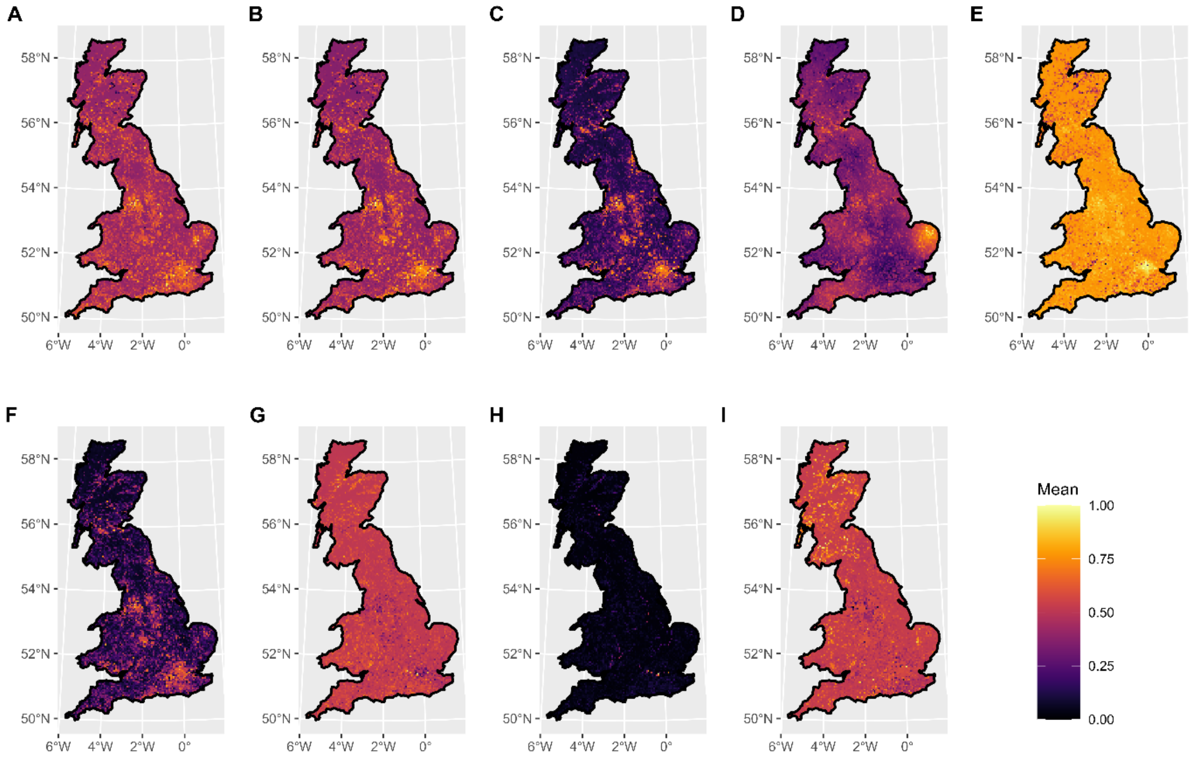

Figure S4. Predicted mean intensity of pest and disease occurrence, rescaled to a range of 0-1, for **A)** *Acer pseudoplatanus*, **B)** *Betula pendula*, **C)** *Fagus sylvatica*, **D)** *Fraxinus excelsior*, **E)** *Quercus robur*, **F)** *Sorbus aucuparia*, **G)** *Picea abies*, **H)** *Picea sitchensis*, and **I)** *Pinus sylvestris*. Predictions were generated from an ISDM using a maximum mesh edge length of 40km. For model covariates, see Table S1.

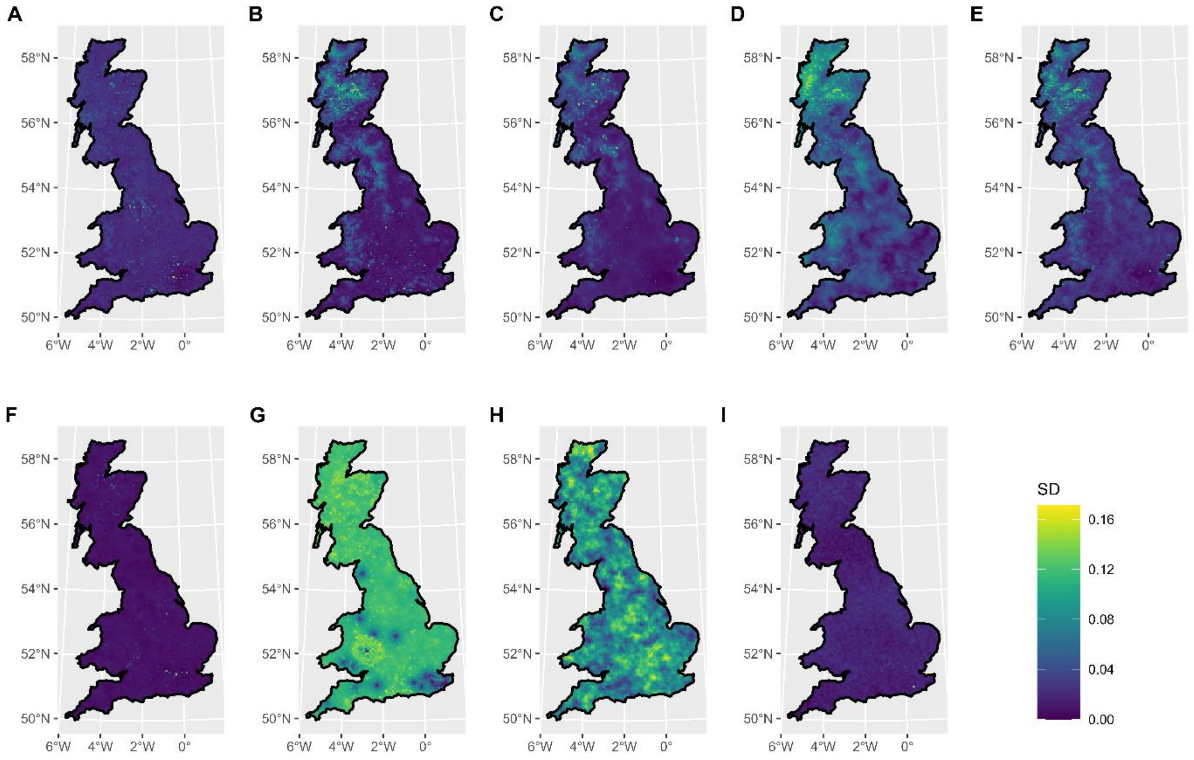

Figure S5. Standard deviation of predicted pest and disease occurrence intensity, rescaled to match the 0-1 scale used for the mean, for **A)** *Acer pseudoplatanus*, **B)** *Betula pendula*, **C)** *Fagus sylvatica*, **D)** *Fraxinus excelsior*, **E)** *Quercus robur*, **F)** *Sorbus aucuparia*, **G)** *Picea abies*, **H)** *Picea sitchensis*, and **I)** *Pinus sylvestris*. Predictions were generated from an ISDM using a maximum mesh edge length of 10km. For model covariates, see Table S1.

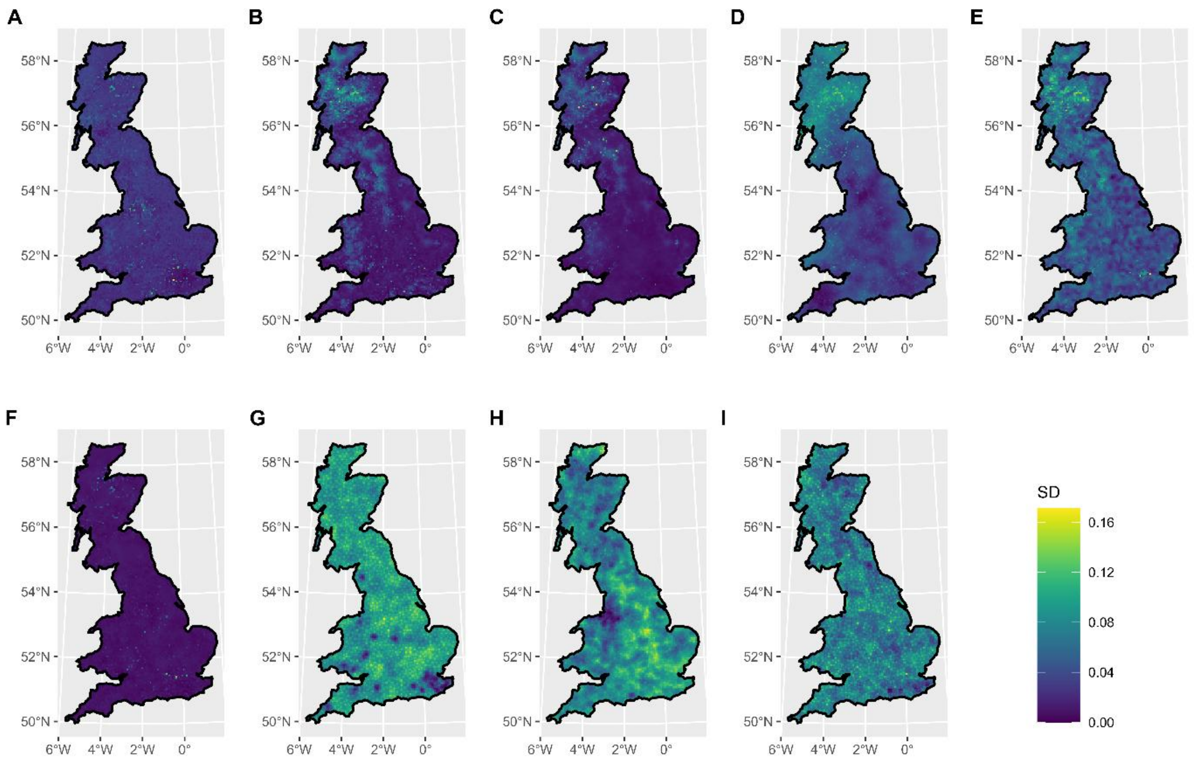

Figure S6. Standard deviation of predicted pest and disease occurrence intensity, rescaled to match the 0-1 scale used for the mean, for **A)** *Acer pseudoplatanus*, **B)** *Betula pendula*, **C)** *Fagus sylvatica*, **D)** *Fraxinus excelsior*, **E)** *Quercus robur*, **F)** *Sorbus aucuparia*, **G)** *Picea abies*, **H)** *Picea sitchensis*, and **I)** *Pinus sylvestris*. Predictions were generated from an ISDM using a maximum mesh edge length of 20km. For model covariates, see Table S1.

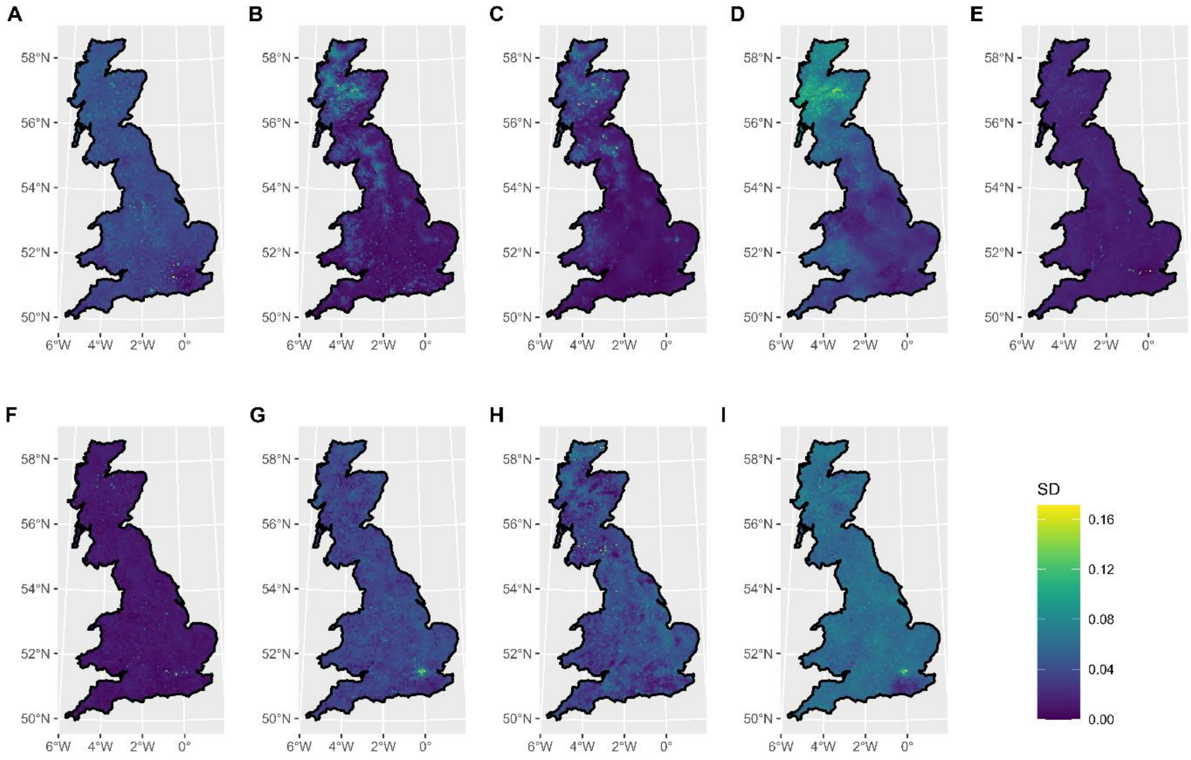

Figure S7. Standard deviation of predicted pest and disease occurrence intensity, rescaled to match the 0-1 scale used for the mean, for **A)** *Acer pseudoplatanus*, **B)** *Betula pendula*, **C)** *Fagus sylvatica*, **D)** *Fraxinus excelsior*, **E)** *Quercus robur*, **F)** *Sorbus aucuparia*, **G)** *Picea abies*, **H)** *Picea sitchensis*, and **I)** *Pinus sylvestris*. Predictions were generated from an ISDM using a maximum mesh edge length of 30km. For model covariates, see Table S1.

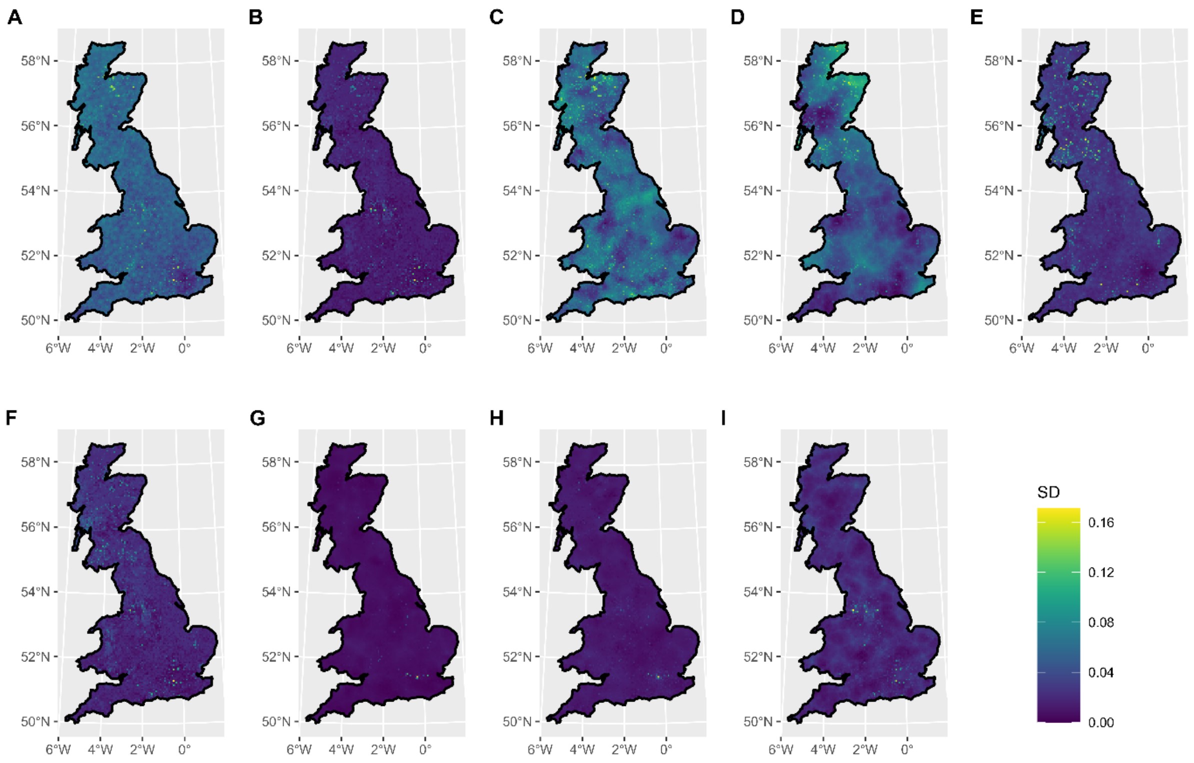

Figure S8. Standard deviation of predicted pest and disease occurrence intensity, rescaled to match the 0-1 scale used for the mean, for **A)** *Acer pseudoplatanus*, **B)** *Betula pendula*, **C)** *Fagus sylvatica*, **D)** *Fraxinus excelsior*, **E)** *Quercus robur*, **F)** *Sorbus aucuparia*, **G)** *Picea abies*, **H)** *Picea sitchensis*, and **I)** *Pinus sylvestris*. Predictions were generated from an ISDM using a maximum mesh edge length of 40km. For model covariates, see Table S1.

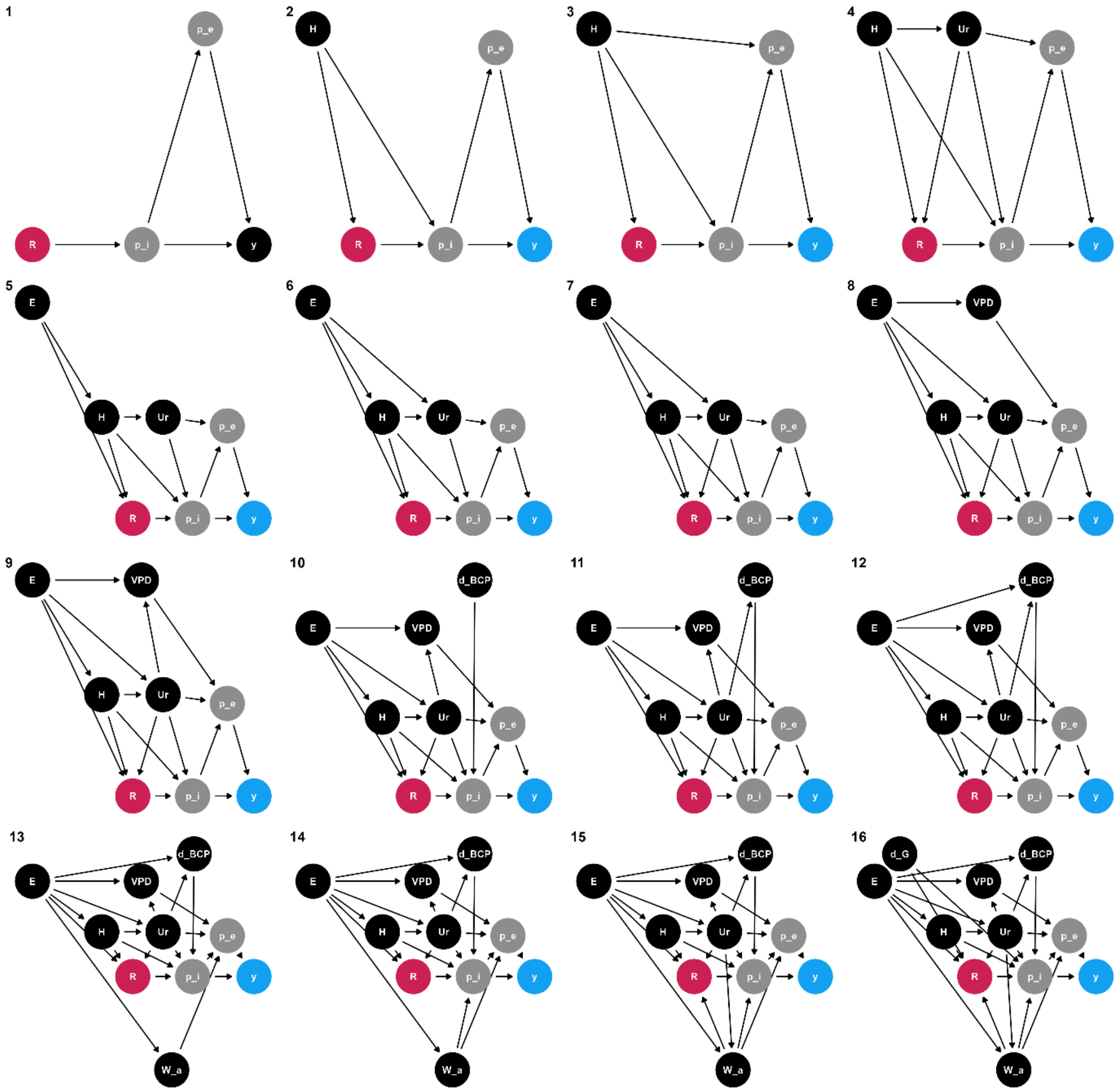

1
p_e R p_i y
2
H p_e R p_i y
3
H p_e R p_i y
4
H Ur p_e R p_i y
5
E H Ur p_e R p_i y
6
E H Ur p_e R p_i y
7
E H Ur p_e R p_i y
8
E VPD H Ur p_e R p_i y
9
E VPD H Ur p_e R p_i y
10
d_BCP E VPD H Ur p_e R p_i y
11
d_BCP E VPD H Ur p_e R p_i y
12
d_BCP E VPD H Ur p_e R p_i y
13
d_BCP E VPD H Ur p_e R p_i y W_a
14
d_BCP E VPD H Ur p_e R p_i y W_a
15
d_BCP E VPD H Ur p_e R p_i y W_a
16
d_G d_BCP E VPD H Ur p_e R p_i y W_a

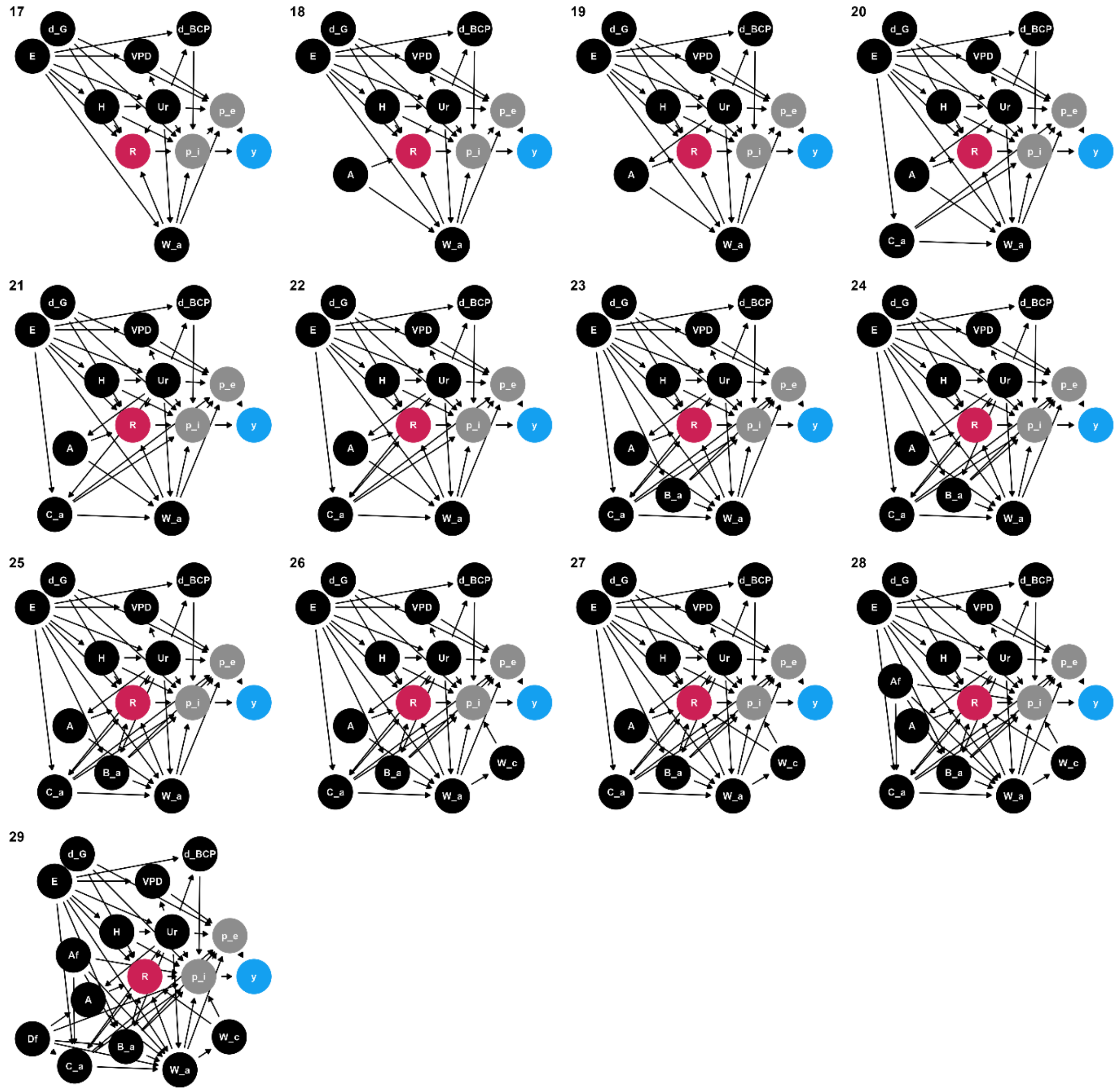

Figure S9. Directed acyclic graphs (DAGs) used to conduct a sensitivity analysis for the effect of recreation on pest and disease occurrence. Each graph represents a set of assumptions about the data generating process; nodes represent arrows, while arrows represent assumed functional links. In all cases, we assumed that pest and disease occurrence (y) is a function of the probability of introduction (p_i) and establishment (p_e), which are latent variables. We added to these assumptions by including one or more additional variables: elevation (E), distance to park/garden (d_G), distance to border control post (d_BCP), vapour pressure deficit (VPD), human population (H), urban area (Ur), afforestation (Af), deforestation (Df), ancient woodland area (A), conifer area (C_a), broadleaf area (B_a), woodland area (W_a), woodland connectivity (W_c), and recreation (R).

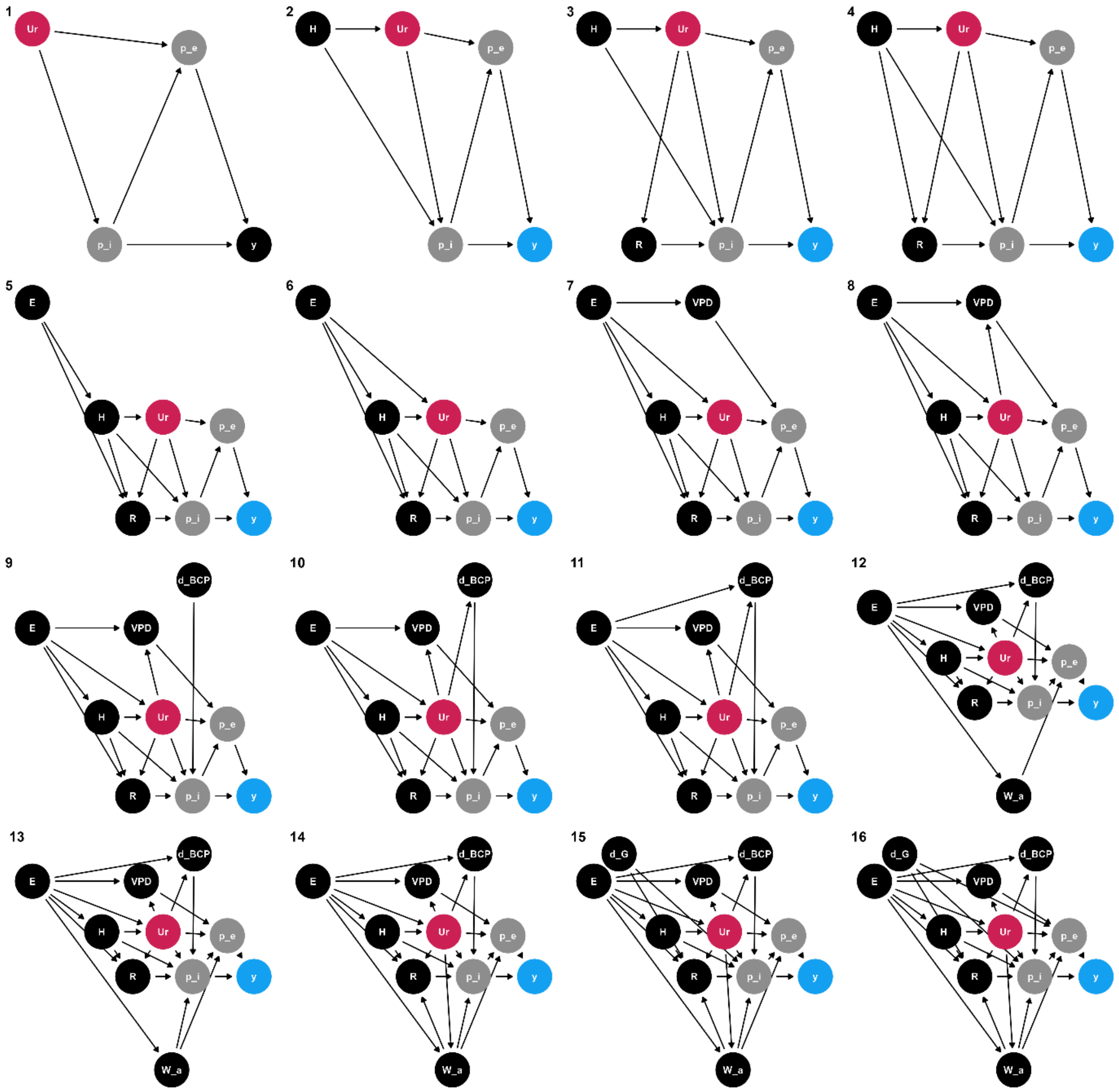
1
Ur
p_e
p_i
y
2
H
3
R
4
5
E
6
7
VPD
8
9
d_BCP
10
11
12
W_a
13
14
15
d_G
16

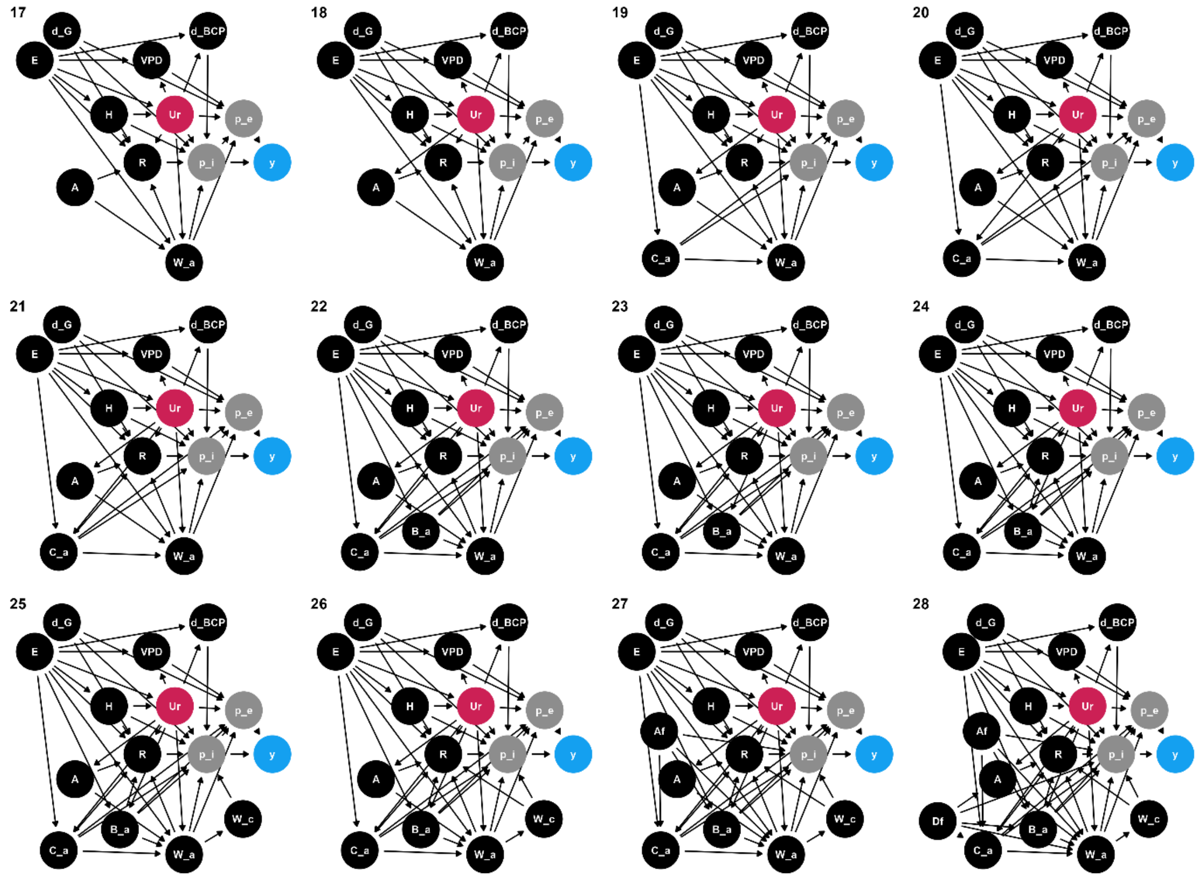

Figure S10. Directed acyclic graphs (DAGs) used to conduct a sensitivity analysis for the effect of urban area on pest and disease occurrence. Each graph represents a set of assumptions about the data generating process; nodes represent arrows, while arrows represent assumed functional links. In all cases, we assumed that pest and disease occurrence (y) is a function of the probability of introduction (p_i) and establishment (p_e), which are latent variables. We added to these assumptions by including one or more additional variables: elevation (E), distance to park/garden (d_G), distance to border control post (d_BCP), vapour pressure deficit (VPD), human population (H), urban area (Ur), afforestation (Af), deforestation (Df), ancient woodland area (A), conifer area (C_a), broadleaf area (B_a), woodland area (W_a), woodland connectivity (W_c), and recreation (R).

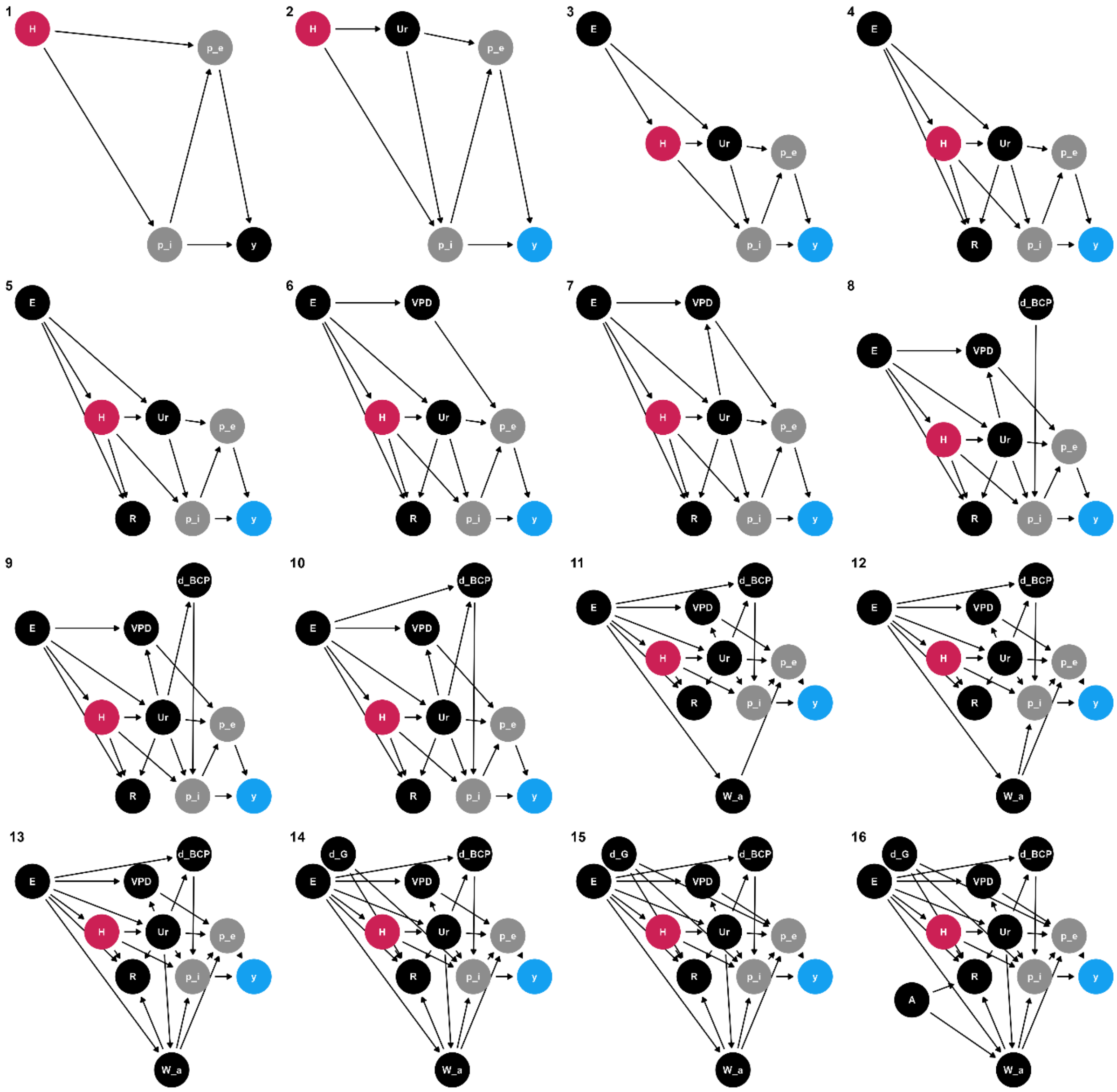
1
2
3
4
5
6
7
8
9
10
11
12
13
14
15
16
H
Ur
E
R
VPD
d_BCP
d_G
A
W_a
p_e
p_i
y

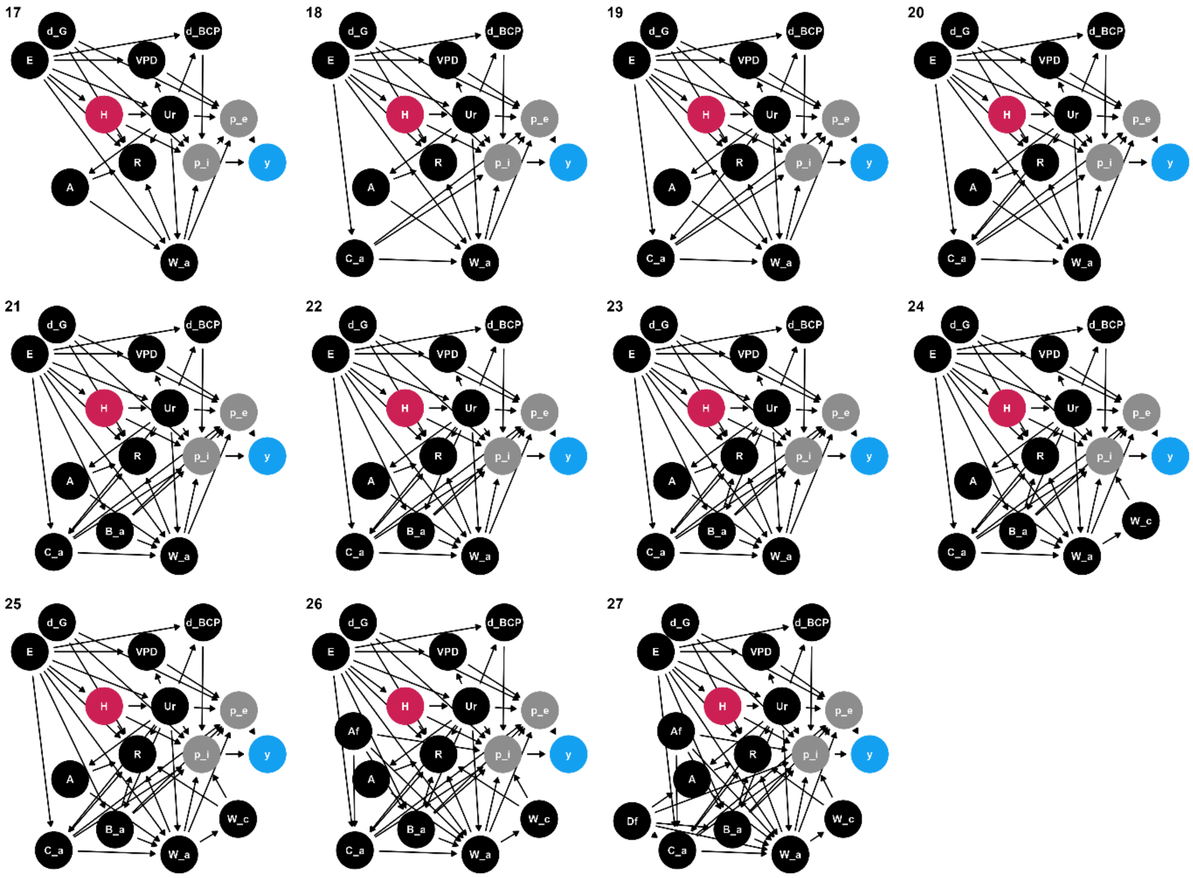

Figure S11. Directed acyclic graphs (DAGs) used to conduct a sensitivity analysis for the effect of human population on pest and disease occurrence. Each graph represents a set of assumptions about the data generating process; nodes represent arrows, while arrows represent assumed functional links. In all cases, we assumed that pest and disease occurrence (y) is a function of the probability of introduction (p_i) and establishment (p_e), which are latent variables. We added to these assumptions by including one or more additional variables: elevation (E), distance to park/garden (d_G), distance to border control post (d_BCP), vapour pressure deficit (VPD), human population (H), urban area (Ur), afforestation (Af), deforestation (Df), ancient woodland area (A), conifer area (C_a), broadleaf area (B_a), woodland area (W_a), woodland connectivity (W_c), and recreation (R).

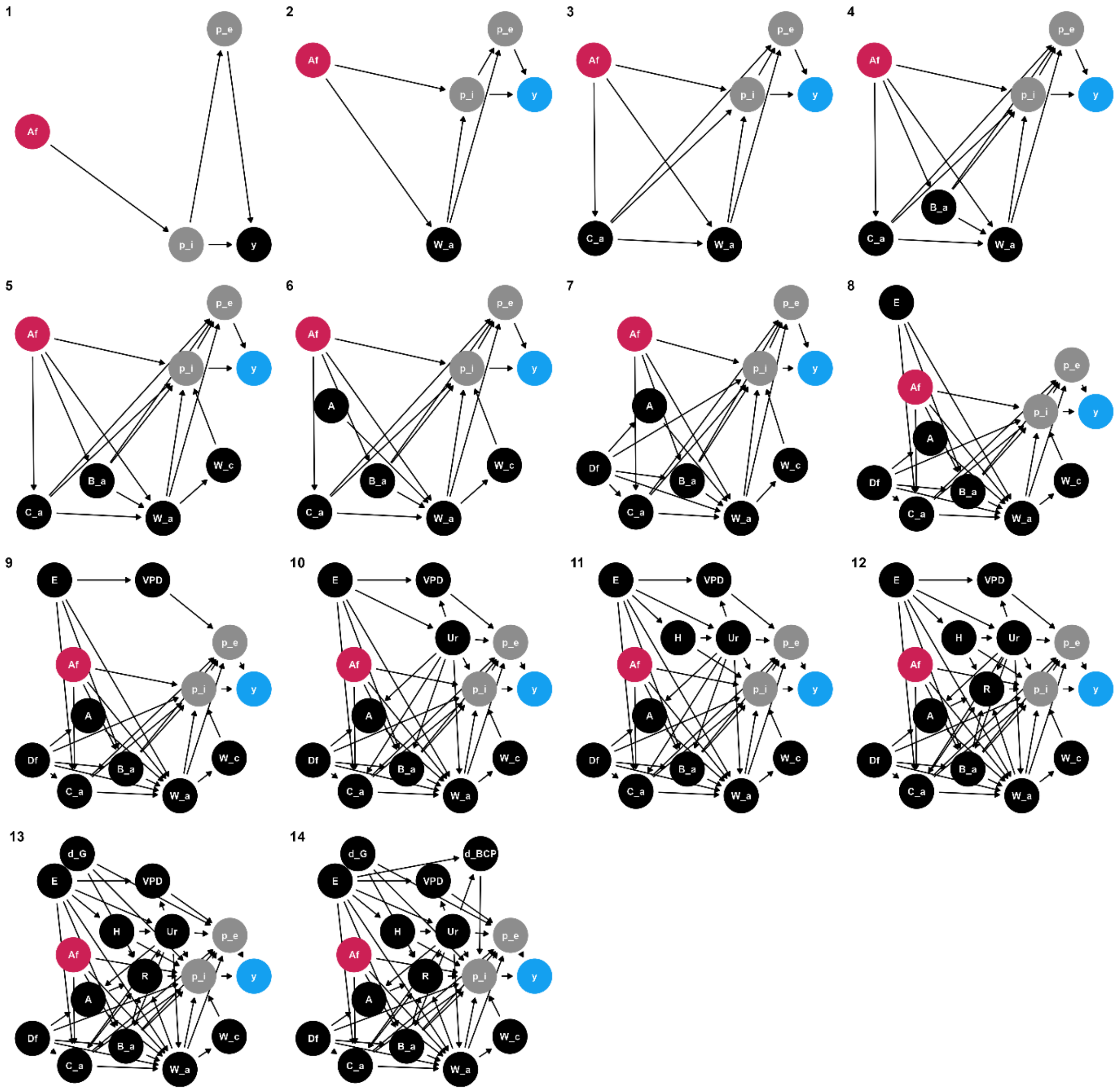

Figure S12. Directed acyclic graphs (DAGs) used to conduct a sensitivity analysis for the effect of afforestation on pest and disease occurrence. Each graph represents a set of assumptions about the data generating process; nodes represent arrows, while arrows represent assumed functional links. In all cases, we assumed that pest and disease occurrence (y) is a function of the probability of introduction (p_i) and establishment (p_e), which are latent variables. We added to these assumptions by including one or more additional variables: elevation (E), distance to park/garden (d_G), distance to border control post (d_BCP), vapour pressure deficit (VPD), human population (H), urban area (Ur), afforestation (Af), deforestation (Df), ancient woodland area (A), conifer area (C_a), broadleaf area (B_a), woodland area (W_a), woodland connectivity (W_c), and recreation (R).

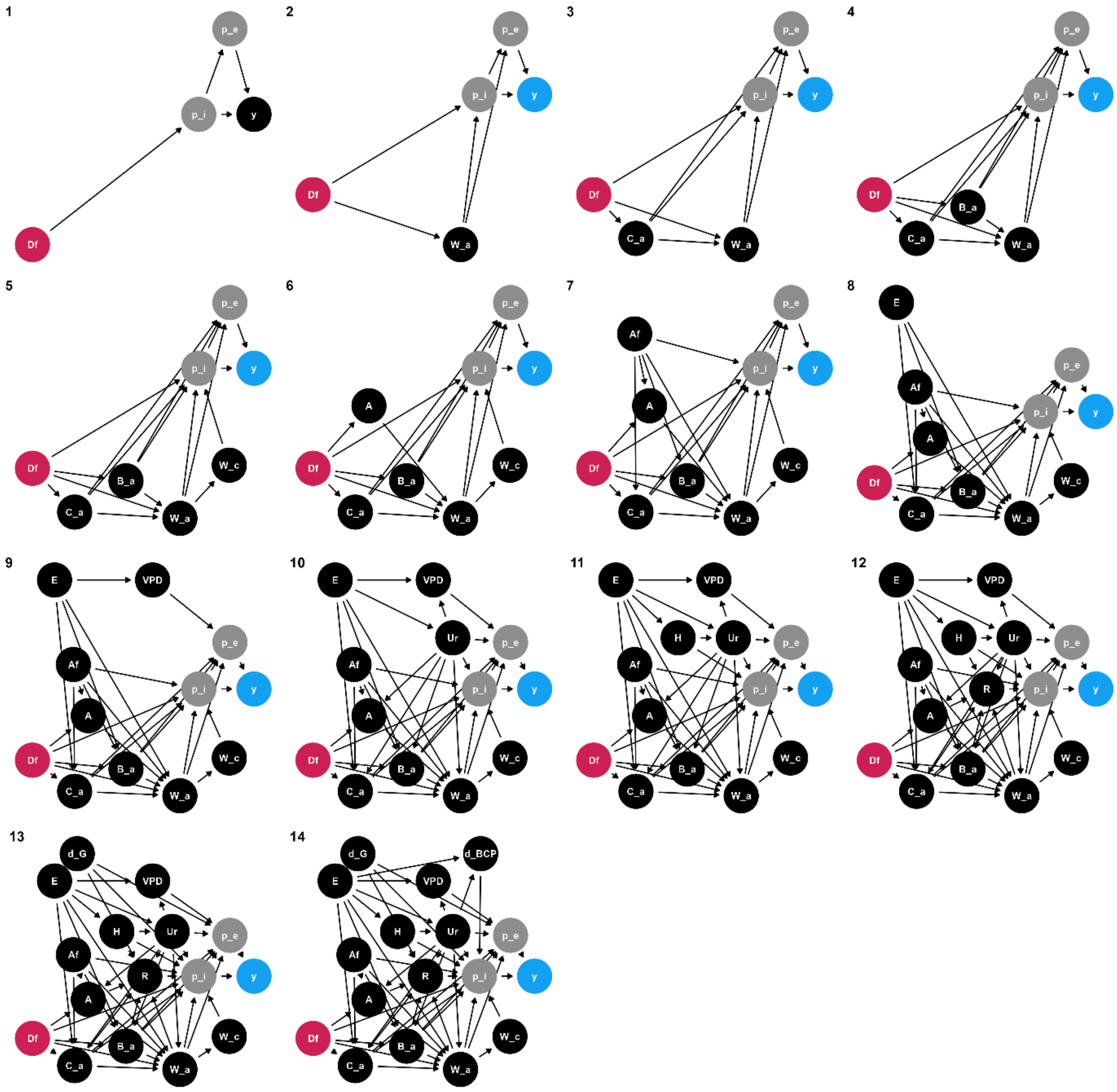

Figure S13. Directed acyclic graphs (DAGs) used to conduct a sensitivity analysis for the effect of deforestation on pest and disease occurrence. Each graph represents a set of assumptions about the data generating process; nodes represent arrows, while arrows represent assumed functional links. In all cases, we assumed that pest and disease occurrence (y) is a function of the probability of introduction (p_i) and establishment (p_e), which are latent variables. We added to these assumptions by including one or more additional variables: elevation (E), distance to park/garden (d_G), distance to border control post (d_BCP), vapour pressure deficit (VPD), human population (H), urban area (Ur), afforestation (Af), deforestation (Df), ancient woodland area (A), conifer area (C_a), broadleaf area (B_a), woodland area (W_a), woodland connectivity (W_c), and recreation (R).

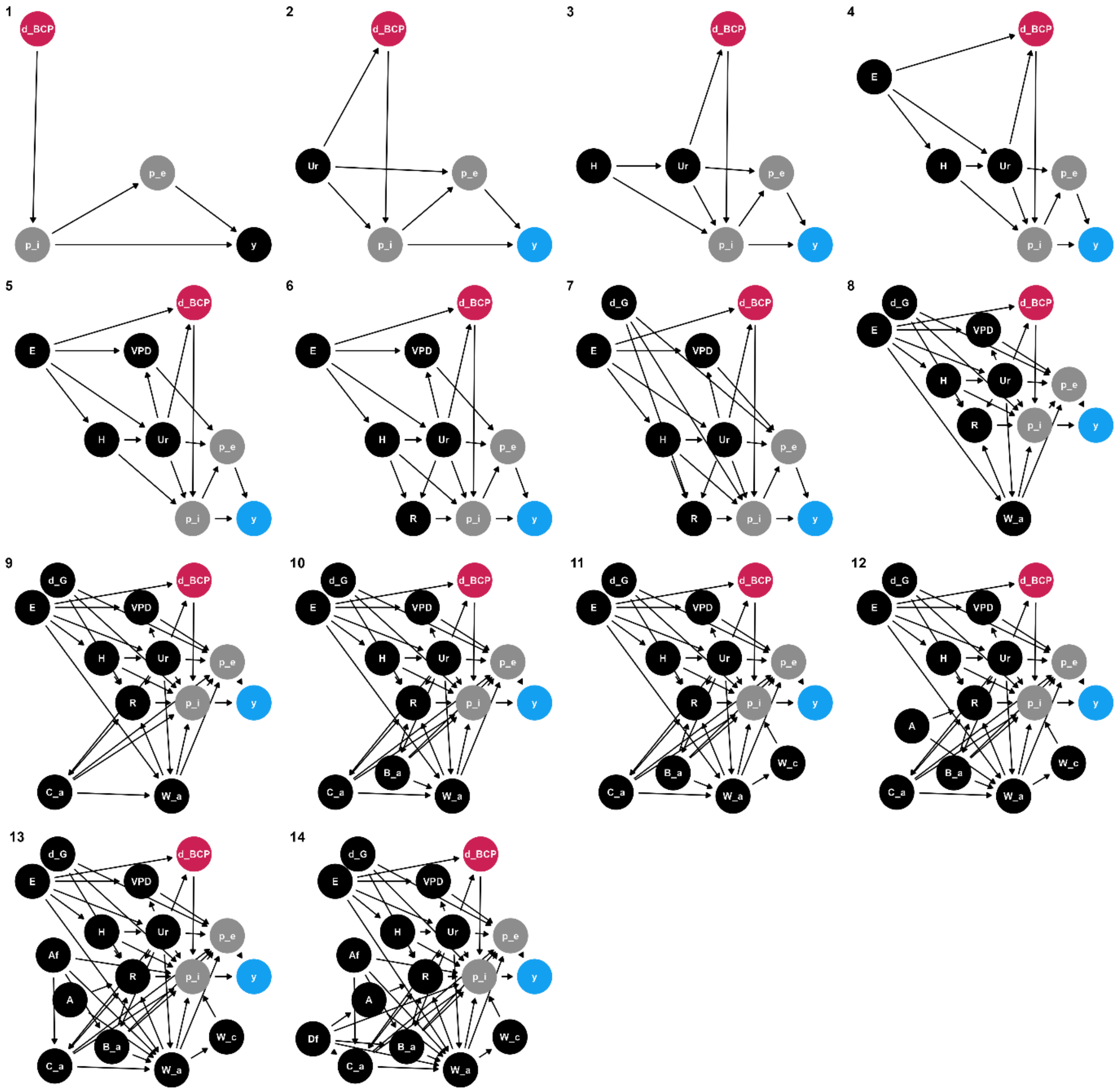

Figure S14. Directed acyclic graphs (DAGs) used to conduct a sensitivity analysis for the effect of distance to border control post on pest and disease occurrence. Each graph represents a set of assumptions about the data generating process; nodes represent arrows, while arrows represent assumed functional links. In all cases, we assumed that pest and disease occurrence (y) is a function of the probability of introduction (p_i) and establishment (p_e), which are latent variables. We added to these assumptions by including one or more additional variables: elevation (E), distance to park/garden (d_G), distance to border control post (d_BCP), vapour pressure deficit (VPD), human population (H), urban area (Ur), afforestation (Af), deforestation (Df), ancient woodland area (A), conifer area (C_a), broadleaf area (B_a), woodland area (W_a), woodland connectivity (W_c), and recreation (R).

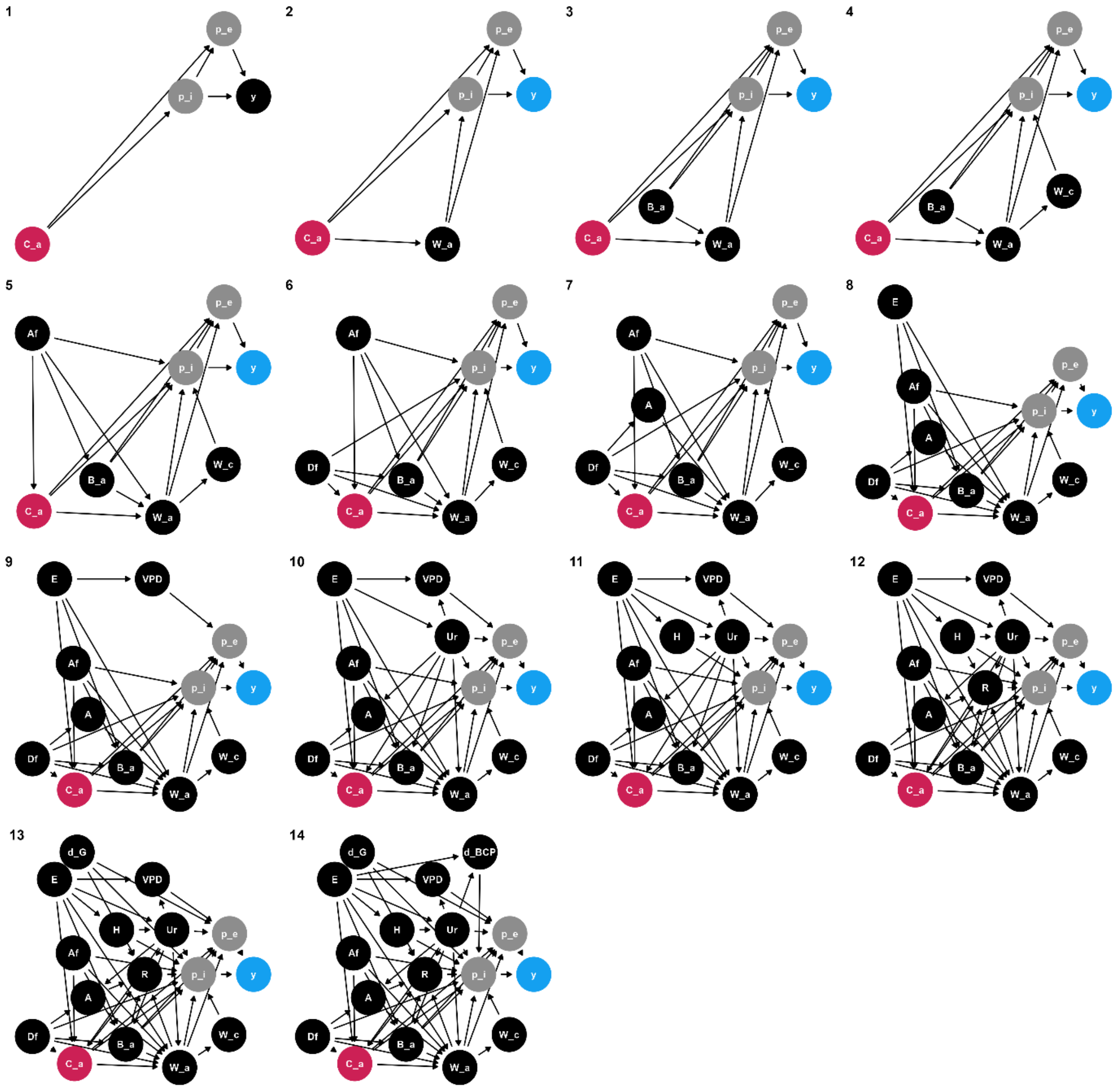

Figure S15. Directed acyclic graphs (DAGs) used to conduct a sensitivity analysis for the effect of conifer area on pest and disease occurrence. Each graph represents a set of assumptions about the data generating process; nodes represent arrows, while arrows represent assumed functional links. In all cases, we assumed that pest and disease occurrence (y) is a function of the probability of introduction (p_i) and establishment (p_e), which are latent variables. We added to these assumptions by including one or more additional variables: elevation (E), distance to park/garden (d_G), distance to border control post (d_BCP), vapour pressure deficit (VPD), human population (H), urban area (Ur), afforestation (Af), deforestation (Df), ancient woodland area (A), conifer area (C_a), broadleaf area (B_a), woodland area (W_a), woodland connectivity (W_c), and recreation (R).

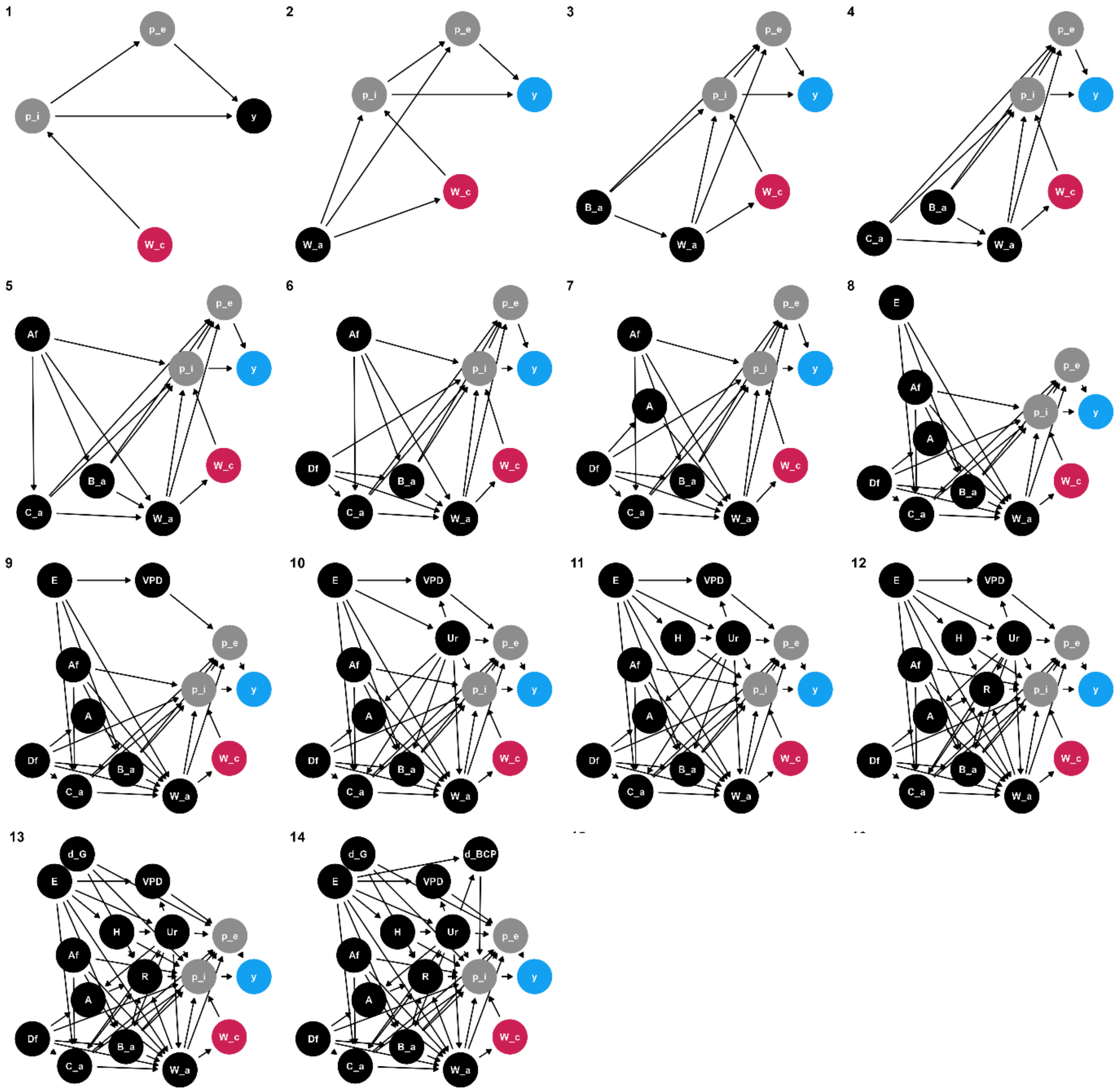

Figure S16. Directed acyclic graphs (DAGs) used to conduct a sensitivity analysis for the effect of woodland connectivity on pest and disease occurrence. Each graph represents a set of assumptions about the data generating process; nodes represent arrows, while arrows represent assumed functional links. In all cases, we assumed that pest and disease occurrence (y) is a function of the probability of introduction (p_i) and establishment (p_e), which are latent variables. We added to these assumptions by including one or more additional variables: elevation (E), distance to park/garden (d_G), distance to border control post (d_BCP), vapour pressure deficit (VPD), human population (H), urban area (Ur), afforestation (Af), deforestation (Df), ancient woodland area (A), conifer area (C_a), broadleaf area (B_a), woodland area (W_a), woodland connectivity (W_c), and recreation (R).

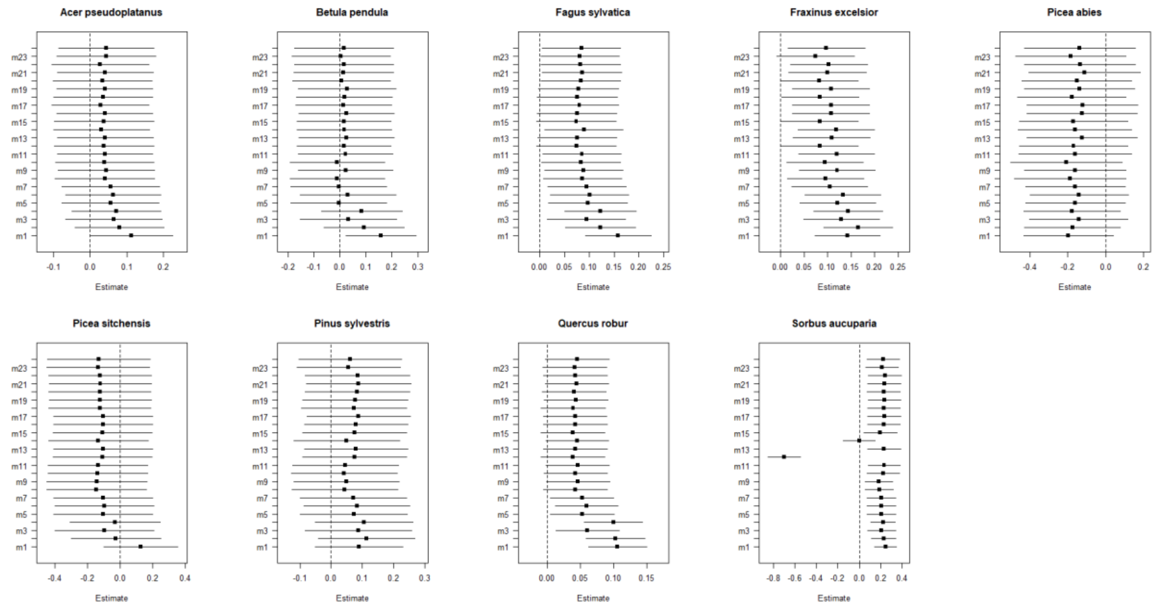

Figure S17. Sensitivity analysis for the effect of weekly recreation on pest and disease occurrence for nine host tree species in mainland Great Britain. Effect estimates were obtained from an ISDM using a maximum edge length of 5km. Points represent posterior mean effects and error bars represent 95% compatibility intervals. Models correspond to unique minimum adjustment sets for DAGs presented in Figure S9.

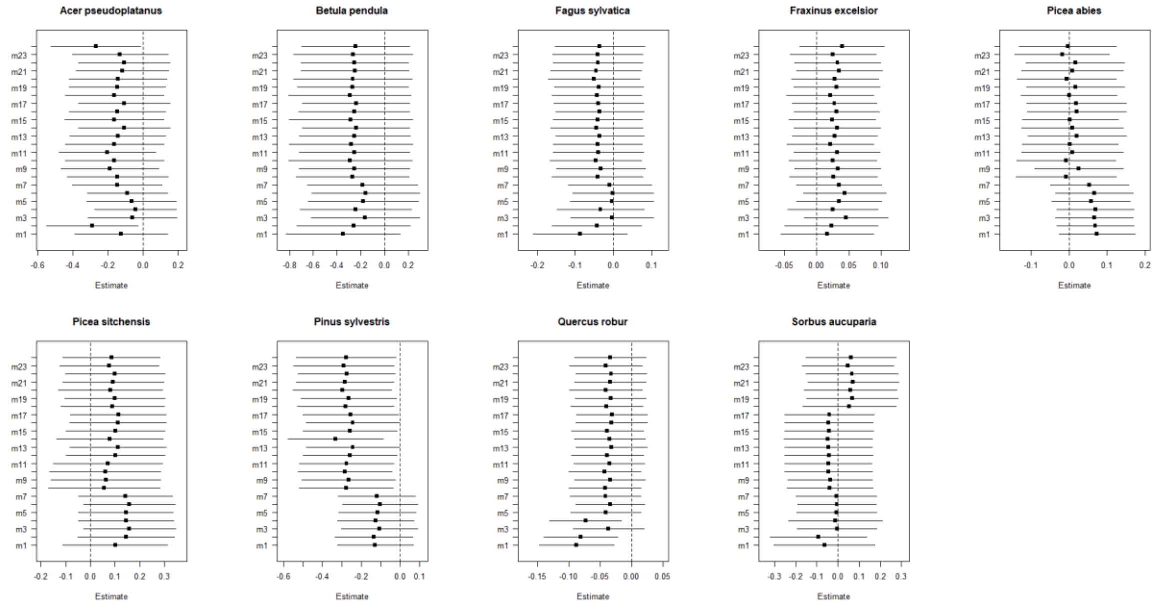

Figure S18. Sensitivity analysis for the effect of yearly recreation on pest and disease occurrence for nine host tree species in mainland Great Britain. Effect estimates were obtained from an ISDM using a maximum edge length of 5km. Points represent posterior mean effects and error bars represent 95% compatibility intervals. Models correspond to unique minimum adjustment sets for DAGs presented in Figure S9.

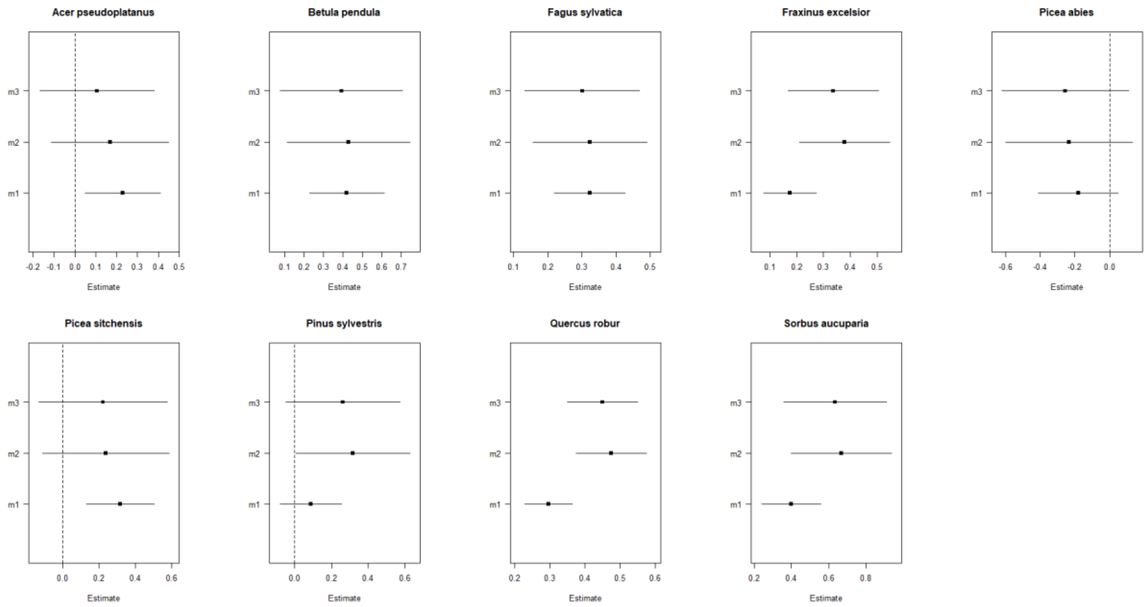

Figure S19. Sensitivity analysis for the effect of urban area on pest and disease occurrence for nine host tree species in mainland Great Britain. Effect estimates were obtained from an ISDM using a maximum edge length of 5km. Points represent posterior mean effects and error bars represent 95% compatibility intervals. Models correspond to unique minimum adjustment sets for DAGs presented in Figure S10.

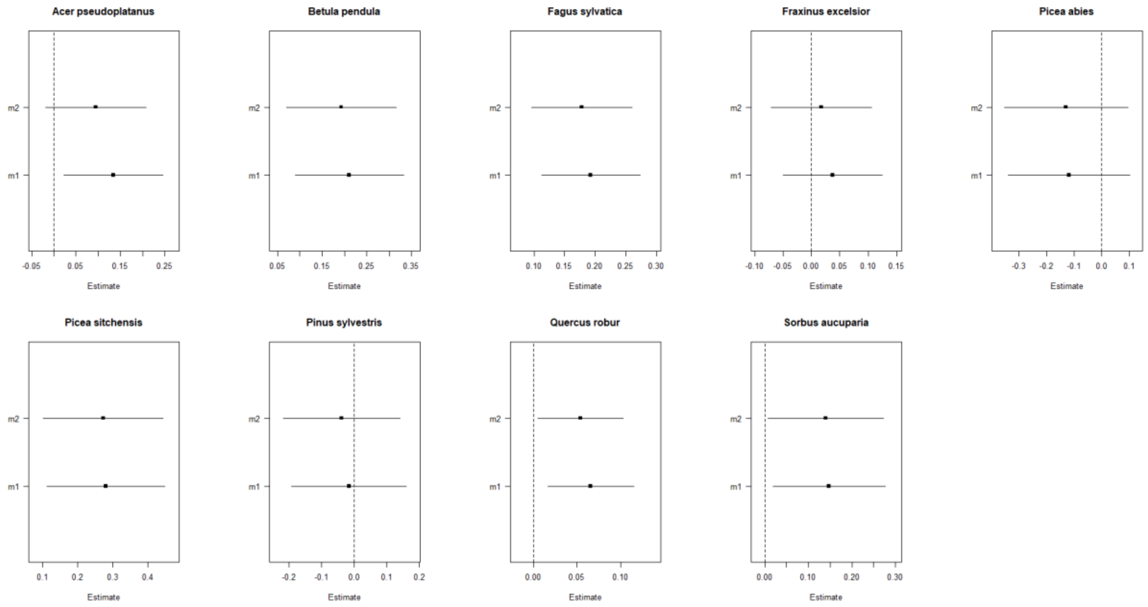

Figure S20. Sensitivity analysis for the effect of human population on pest and disease occurrence for nine host tree species in mainland Great Britain. Effect estimates were obtained from an ISDM using a maximum edge length of 5km. Points represent posterior mean effects and error bars represent 95% compatibility intervals. Models correspond to unique minimum adjustment sets for DAGs presented in Figure S11.

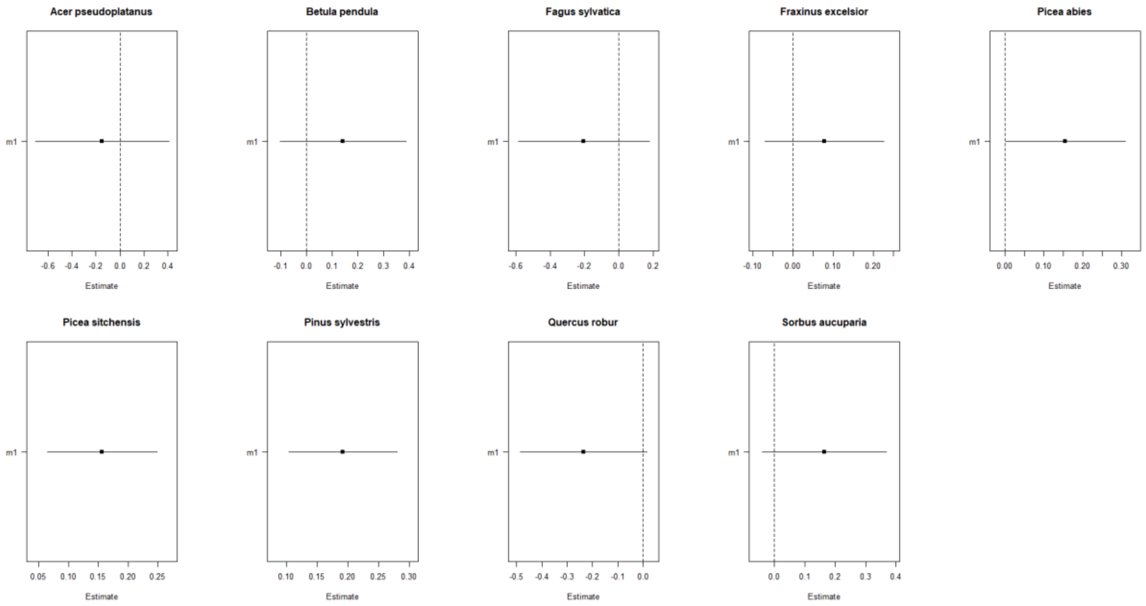

Figure S21. Sensitivity analysis for the effect of afforestation on pest and disease occurrence for nine host tree species in mainland Great Britain. Effect estimates were obtained from an ISDM using a maximum edge length of 5km. Points represent posterior mean effects and error bars represent 95% compatibility intervals. Models correspond to unique minimum adjustment sets for DAGs presented in Figure S12.

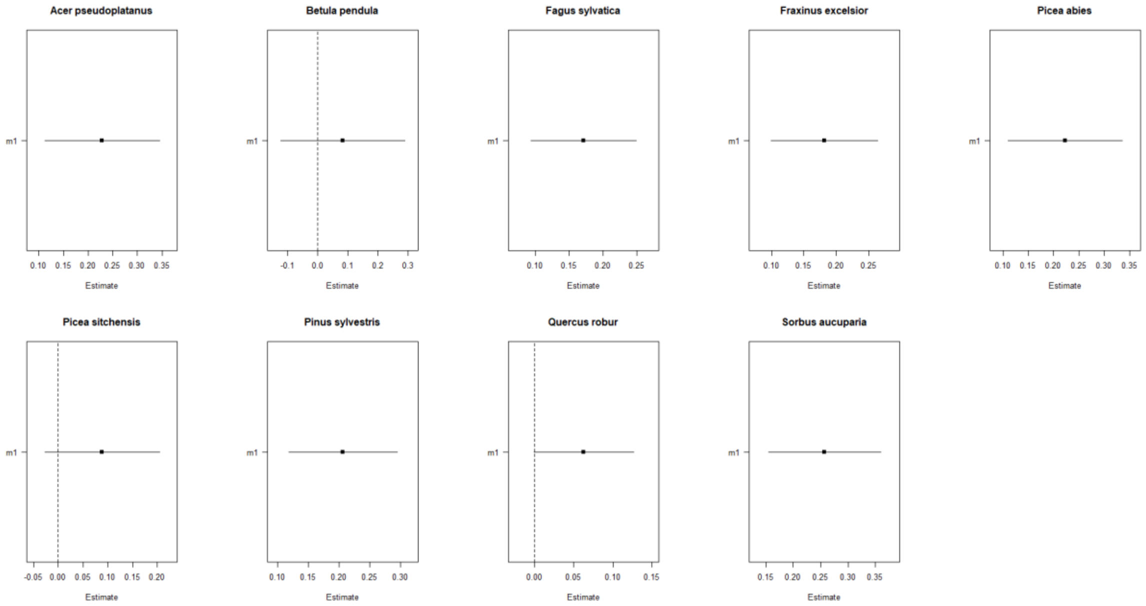

Figure S22. Sensitivity analysis for the effect of deforestation on pest and disease occurrence for nine host tree species in mainland Great Britain. Effect estimates were obtained from an ISDM using a maximum edge length of 5km. Points represent posterior mean effects and error bars represent 95% compatibility intervals. Models correspond to unique minimum adjustment sets for DAGs presented in Figure S13.

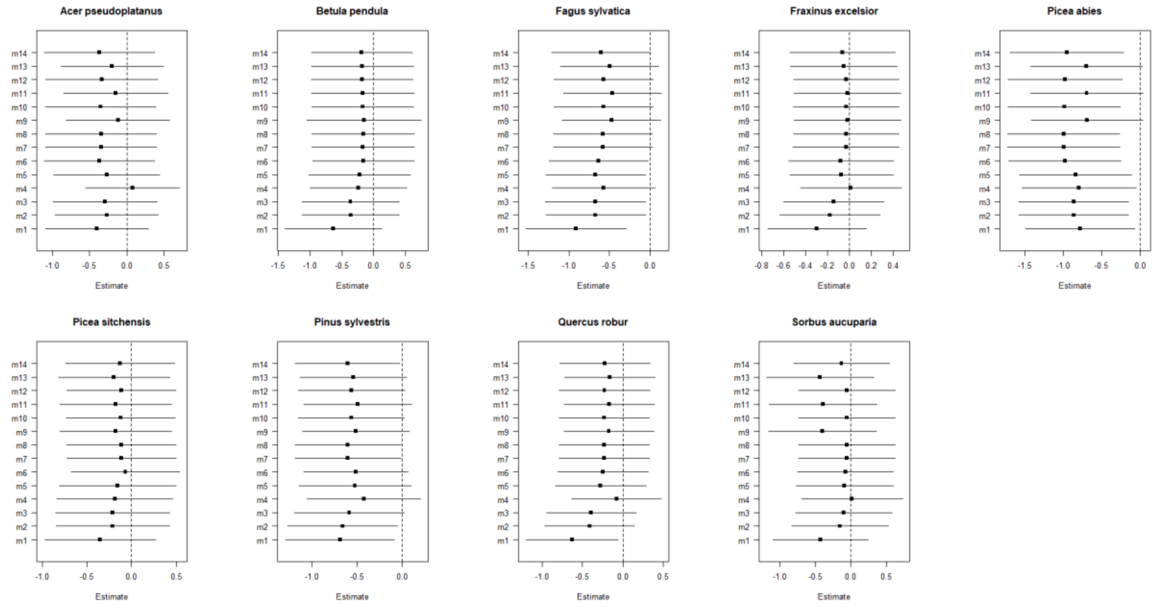

Figure S23. Sensitivity analysis for the effect of distance to border control post on pest and disease occurrence for nine host tree species in mainland Great Britain. Effect estimates were obtained from an ISDM using a maximum edge length of 5km. Points represent posterior mean effects and error bars represent 95% compatibility intervals. Models correspond to unique minimum adjustment sets for DAGs presented in Figure S14.

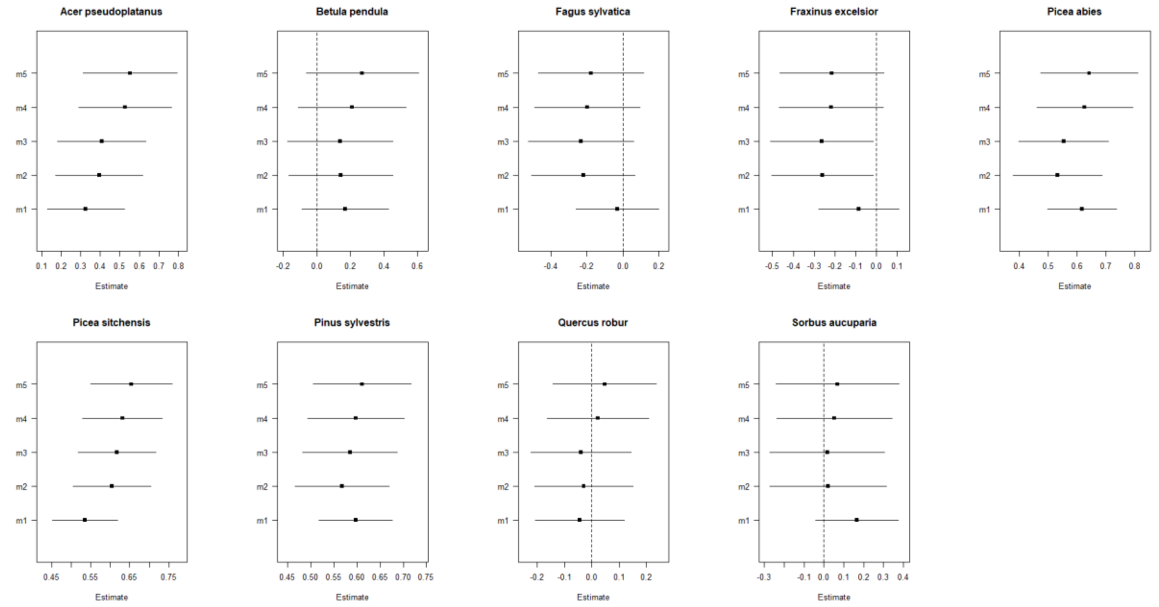

Figure S24. Sensitivity analysis for the effect of conifer area on pest and disease occurrence for nine host tree species in mainland Great Britain. Effect estimates were obtained from an ISDM using a maximum edge length of 5km. Points represent posterior mean effects and error bars represent 95% compatibility intervals. Models correspond to unique minimum adjustment sets for DAGs presented in Figure S15.

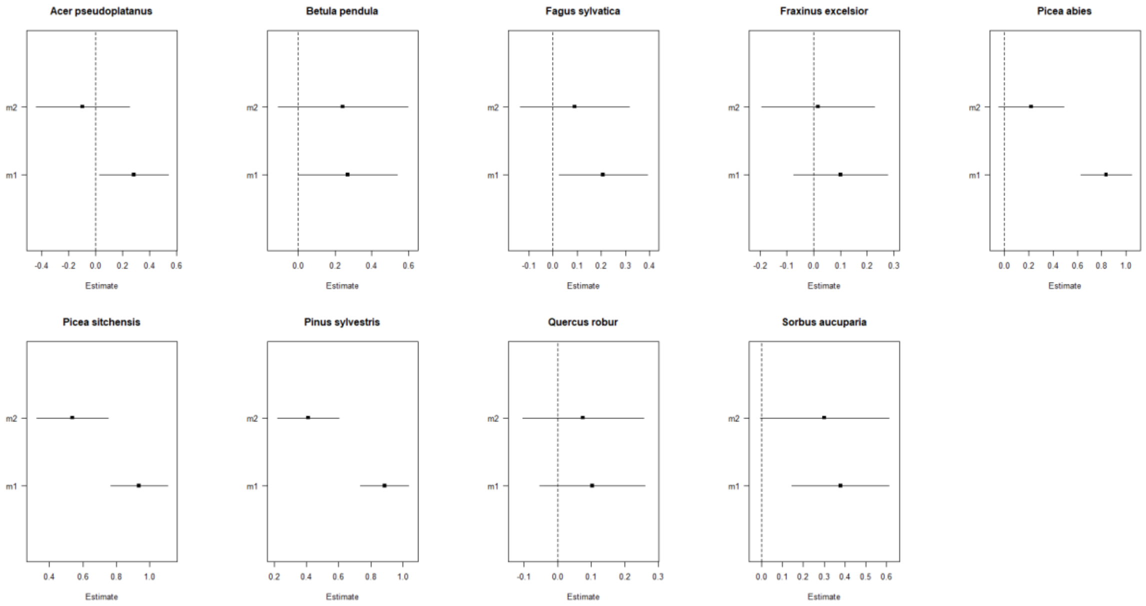

Figure S25. Sensitivity analysis for the effect of woodland connectivity on pest and disease occurrence for nine host tree species in mainland Great Britain. Effect estimates were obtained from an ISDM using a maximum edge length of 5km. Points represent posterior mean effects and error bars represent 95% compatibility intervals. Models correspond to unique minimum adjustment sets for DAGs presented in Figure S16.

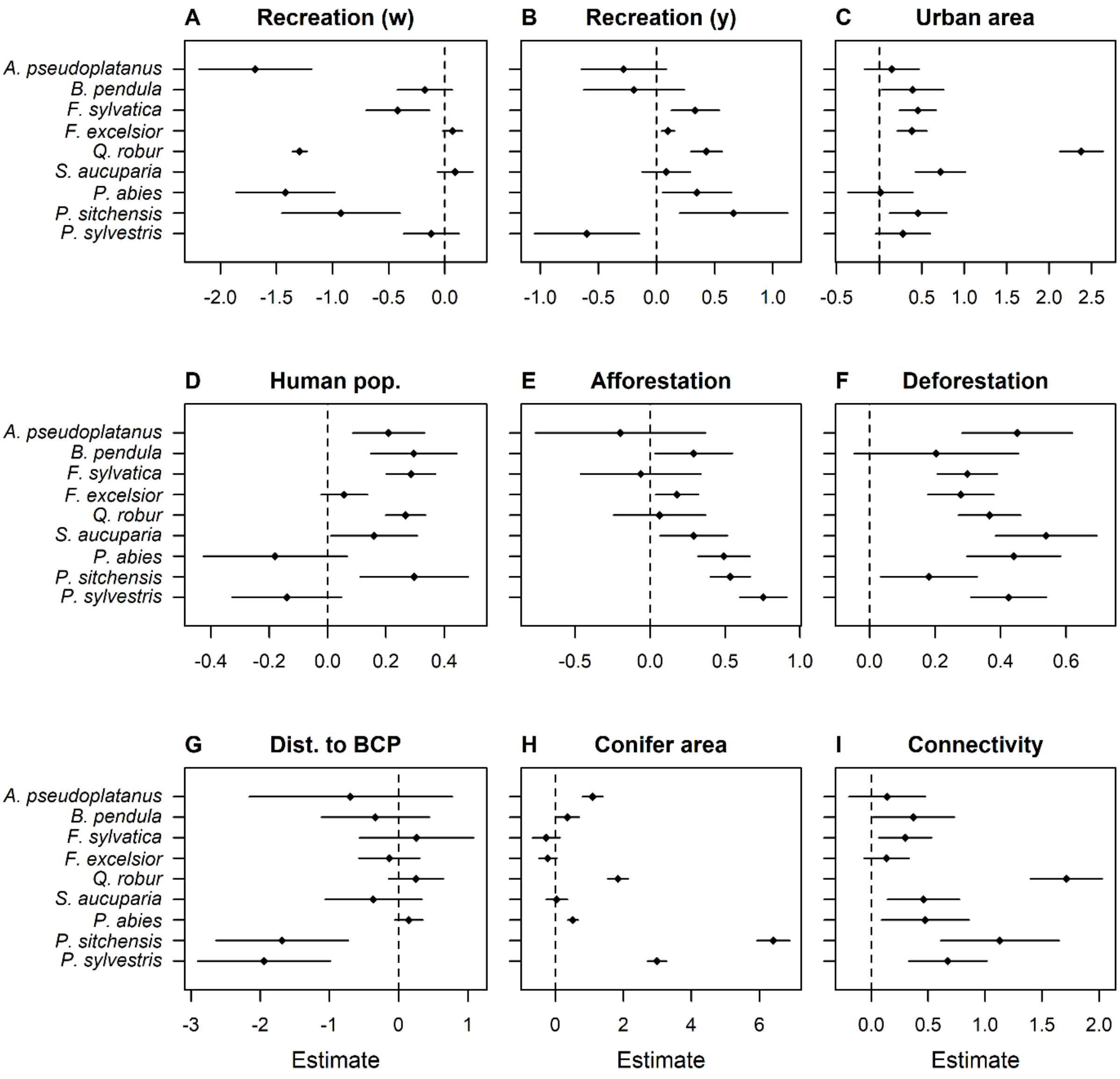

Figure S26. Posterior mean effect (± 95% compatibility interval) of nine focal variables on pest and disease occurrence for nine host tree species. Effect estimates were produced by ISDMs using a maximum edge length of 30km. Dist. to BCP = distance to border control post.
